# Development and evaluation of a milk protein transcript depletion method for differential transcriptome analysis in mammary gland tissue

**DOI:** 10.1186/s12864-019-5781-3

**Published:** 2019-05-22

**Authors:** Johanna Brodhagen, Rosemarie Weikard, Ulrike Thom, Annika Heimes, Juliane Günther, Frieder Hadlich, Holm Zerbe, Wolfgang Petzl, Marie M. Meyerholz, Martina Hoedemaker, Hans-Joachim Schuberth, Susanne Engelmann, Christa Kühn

**Affiliations:** 1 Leibniz Institute for Farm Animal Biology (FBN), Institute of Genome Biology, 18196 Dummerstorf, Germany; 2 Clinic for Ruminants with Ambulatory and Herd Health Services, Centre for Clinical Veterinary Medicine, Ludwig-Maximilians-University Munich, 85764 Oberschleissheim, Germany; 3 Clinic for Cattle, University of Veterinary Medicine, Foundation, 30173 Hannover, Germany; 4 Immunology Unit, University of Veterinary Medicine, Foundation, 30559 Hannover, Germany; 5 Institute for Microbiology, Technical University Braunschweig, 38106 Braunschweig, Germany; 6 Microbial Proteomics, Helmholtz Centre for Infection Research, 38124 Braunschweig, Germany; 7 Agricultural and Environmental Faculty, University Rostock, 18059 Rostock, Germany

**Keywords:** RNA depletion, RNA-sequencing, Mammary gland, Lactation, Mastitis

## Abstract

**Background:**

In the mammary gland transcriptome of lactating dairy cows genes encoding milk proteins are highly abundant, which can impair the detection of lowly expressed transcripts and can bias the outcome in global transcriptome analyses. Therefore, the aim of this study was to develop and evaluate a method to deplete extremely highly expressed transcripts in mRNA from lactating mammary gland tissue.

**Results:**

Selective RNA depletion was performed by hybridization of antisense oligonucleotides targeting genes encoding the caseins (*CSN1S1, CSN1S2, CSN2* and *CSN3*) and whey proteins (*LALBA* and *PAEP*) within total RNA followed by RNase H-mediated elimination of the respective transcripts. The effect of the RNA depletion procedure was monitored by RNA sequencing analysis comparing depleted and non-depleted RNA samples from *Escherichia coli* (*E. coli*) challenged and non-challenged udder tissue of lactating cows in a proof of principle experiment. Using RNase H-mediated RNA depletion, the ratio of highly abundant milk protein gene transcripts was reduced in all depleted samples by an average of more than 50% compared to the non-depleted samples. Furthermore, the sensitivity for discovering transcripts with marginal expression levels and transcripts not yet annotated was improved. Finally, the sensitivity to detect significantly differentially expressed transcripts between non-challenged and challenged udder tissue was increased without leading to an inadvertent bias in the pathogen challenge-associated biological signaling pathway patterns.

**Conclusions:**

The implementation of selective RNase H-mediated RNA depletion of milk protein gene transcripts from the mammary gland transcriptome of lactating cows will be highly beneficial to establish comprehensive transcript catalogues of the tissue that better reflects its transcriptome complexity.

**Electronic supplementary material:**

The online version of this article (10.1186/s12864-019-5781-3) contains supplementary material, which is available to authorized users.

## Background

RNA sequencing (RNA-Seq) has revolutionized the study of whole transcriptomes in cells and tissues and has opened a new horizon for the understanding of global gene expression by providing fundamental new insights into the structural organization and functional regulation of the genomes at transcriptional level. Molecular cataloguing of cell- and tissue-specific transcriptome elements is essential for the identification and functional annotation of regulatory features. These are important to understand physiological changes and molecular mechanisms in an organism in response to environmental challenges, during development and under disease-associated conditions.

Bovine mastitis is an inflammatory disease of the mammary gland mainly due to bacterial infection [1, 2]. It affects welfare and health of the cows, resulting in financial losses due to reduced performance, increased treatment costs and animal losses, especially in dairy farming [3–7]. Cows often suffer from mastitis during early lactation, highlighting this period to be important for the investigation of individual mastitis susceptibility [8, 9]. However, the molecular regulatory mechanisms involved in different mastitis susceptibility of cows are complex and not yet clarified in detail. Transcriptomic studies using RNA-Seq can help to identify the gene clusters or networks that are involved in the regulation of processes affecting mastitis susceptibility and incidence in the mammary gland. Transcriptome profiling and the identification of a comprehensive transcript catalogue of the mammary tissue might be compromised due to the fact that at the stage of lactation the transcriptome of the mammary gland is overrepresented by highly abundant transcripts of genes encoding milk proteins, such as genes from the casein gene cluster (*CSN2, CSN3, CSN1S1, CSN1S2*) and whey protein genes, progestogen-associated endometrial protein gene (*PAEP*) encoding ß lactoglobulin and α lactalbumin (*LALBA*) [10, 11]. Transcriptome studies in mammary tissue and milk cells of dairy cows have confirmed this transcriptional pattern and reported that the percentage of milk protein gene transcripts can account for up to 70% of all transcripts expressed in this tissue, which may impair the detection of transcripts of genes with a marginal expression level at a given sequencing depth [12]. As a consequence, transcripts expressed at a lower level compared to protein coding genes, e.g., long noncoding RNAs, which may be involved in regulatory processes of immune defence in the lactating udder during infection [13–15], will possibly not be recognized by global transcriptome analysis.

In order to be able to identify even rare transcripts accurately and reliably in the lactating mammary gland transcriptome, a high level of sequencing depth must be achieved, but this ultimately leads to higher sequencing costs. The aim of our study was therefore to develop a reliable, cost-efficient method that reduces the proportion of high-frequency transcripts in mRNA from bovine mammary gland tissue in order to be able to establish a comprehensive catalogue of mammary gland transcripts. Using this method, an improvement of detection sensitivity of transcripts with marginal expression levels should be achieved while simultaneously the costs for deep transcriptome profiling of mammary gland from lactating cows via RNA-Seq are reduced.

There are two predominant techniques underlying the different RNA depletion procedures and commercially available kits applied to reduce the prevalence of highly abundant genes, such as globin RNA in blood and ribosomal and mitochondrial RNA fractions in total RNA from various tissues. In the first step, both strategies rely on hybridization of gene-specific complementary oligonucleotides to the targeted gene sequences in the total RNA. The difference between these techniques is in eliminating the unwanted targeted genes from the pool of total RNA sequences. In the second step, therefore one strategy is based on capturing of the RNA:DNA duplexes using the paramagnetic bead technology. In the alternative strategy, the RNA:DNA hybrids are degraded by RNAse H so that the targeted sequences are no longer available for subsequent applications such as polyA+ selection. A cross-site comparative study with commercially available rRNA depletion kits including kits based on the capture of rRNA by complementary oligonucleotides coupled to beads and also kits based on the hybridization of rRNA with antisense DNA oligonucleotides followed by degradation of RNA:DNA hybrids with RNase H, showed that although there were differences between the underlying rRNA depletion chemistries, all tested kits were able to successfully remove a significant amount of rRNA in library preparations [16]. All kits were able to remove ribosomal RNA to below 20%, but in comparison, the kits that degraded the rRNA by RNase H treatment showed more consistent results than kits that used the bead-based capture method for rRNA depletion. Furthermore, Adiconis et al. [17] and Herbert et al. [16] found that the RNase H-mediated method performed best for rRNA depletion in case of low quality RNA.

In our study we developed an RNase H-mediated RNA depletion approach targeting highly expressed milk protein genes, which was validated experimentally on total RNA isolated from *E. coli* challenged and non-challenged mammary gland tissue of three lactating Holstein-Friesian dairy cows. To monitor the performance and efficiency of this targeted RNA depletion protocol, a comparative RNA-Seq analysis was carried out on depleted and non-depleted mammary gland RNA samples.

## Results and discussion

### Optimization of the RNAse H-mediated RNA depletion procedure for milk protein genes highly prevalent in the mammary gland transcriptome

Targeted RNA removal of highly abundant transcripts prior to RNA-Seq has been successfully introduced into commercial library preparation kits to deplete globin RNA in blood and ribosomal and mitochondrial RNA fractions in total RNA from various tissue types [18–21].

**Fig. 1:**
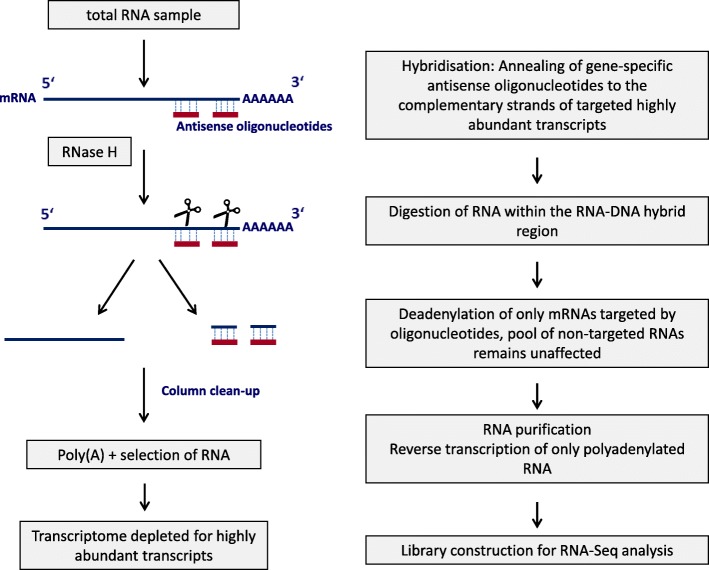
Workflow for RNase H- mediated RNA depletion of highly abundant transcripts

Focusing on the removal of highly abundant milk protein transcripts in the total RNA pool extracted from mammary gland samples of lactating cows, we have developed an RNase H-mediated RNA depletion method based on sequence-specific anti-sense oligonucleotides targeting milk protein genes. The final methodological procedure is illustrated in Fig. 1.

Prior to applying this RNA depletion strategy in a proof of principle experiment on RNA samples from pathogen-challenged and non-challenged udder tissue of lactating cows, several technical optimization steps were performed and monitored by comparative RT-qPCR analysis of depleted and non-depleted RNA samples. Total RNA extracted from udder samples of two lactating cows was used to analyze the effect of modifications in the RNA depletion protocol.

In the initial experiment we adjusted the ratio of antisense oligonucleotides in the oligonucleotide depletion mix according to the abundance of the targeted milk protein genes in lactating mammary gland as retrieved from literature data [10]. Since RT-qPCR monitoring of the depletion treatment showed a smaller depletion effect for *LALBA*, *CSN1S1* and *PAEP* compared to *CSN3*, *CSN2* and *CSN1S2*, we modified the hybridization assay conditions and replaced the antisense oligonucleotide sets for *LALBA*, *CSN1S1* and *PAEP* with others. These modifications established in RNA depletion variant A improved the RNA depletion efficiency only slightly. In addition, the effect of the RNA depletion procedure itself (RNA treatment with buffer instead of antisense oligonucleotides) was also monitored.

**Fig. 2:**
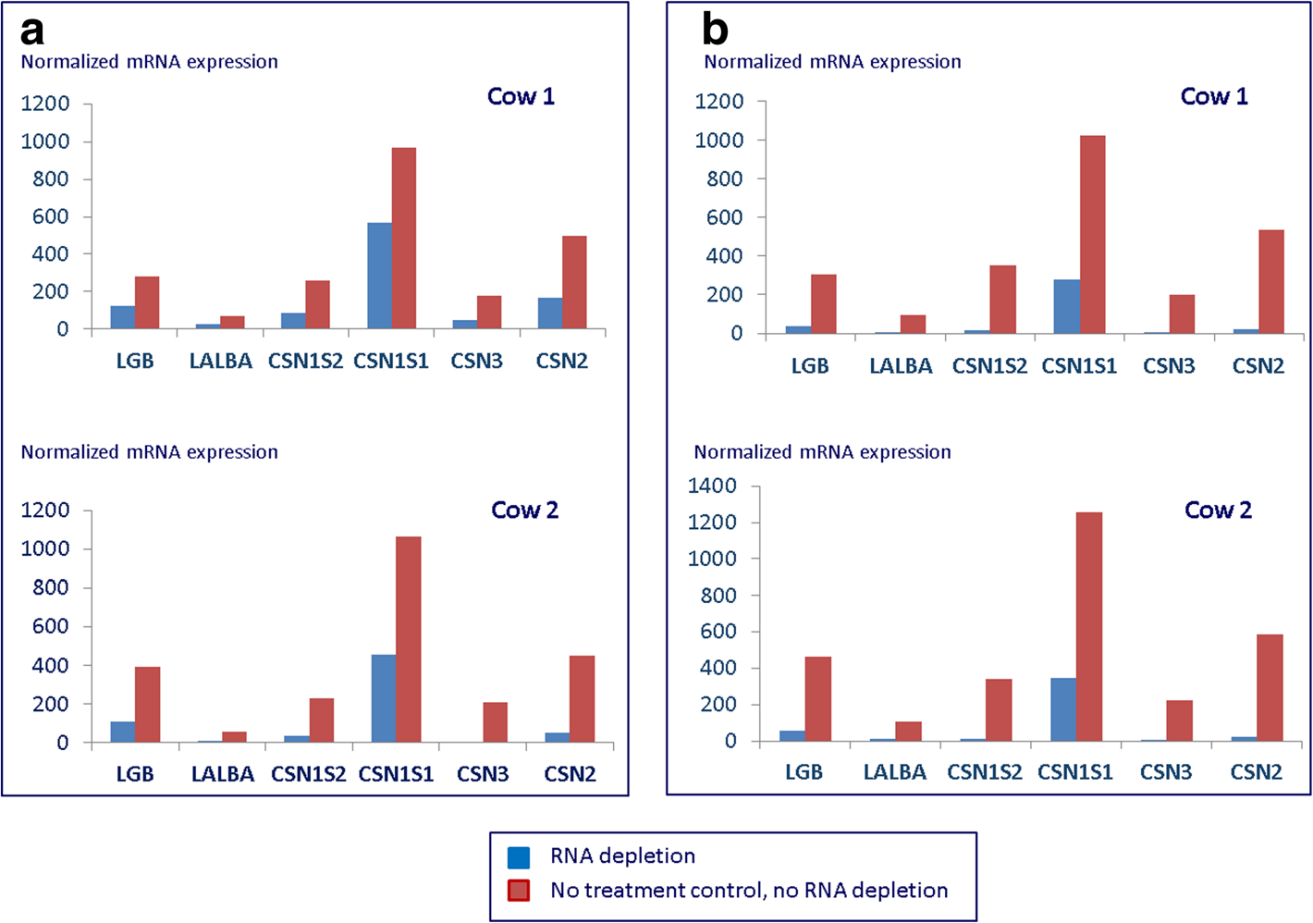
Effect of RNase H-mediated depletion of highly abundant transcripts on targeted genes in the mammary gland of lactating cows monitored by RT-qPCR analysis. **a** Ratio of antisense oligonucleotides in the RNA depletion mix according to the abundance of targeted genes as expected from other studies and **b** Higher, and identical concentration of antisense oligonucleotides in the RNA depletion mix (see Materials and Methods)

Finally, the concentration of the respective oligonucleotides in the depletion mix was modified and adjusted to the same equimolar level for all oligonucleotides for RNA depletion variant B. In the end, the results of RT-qPCR analysis of depleted and non-depleted samples revealed that the mRNA abundance of targeted milk protein genes was reduced by 30 to 90% in the depleted samples depending on the specific gene targeted and the experimental conditions applied. Examples of RT-qPCR evaluation for the two methodological variants A and B used in our follow-up study on RNA samples from pathogen-challenged and non-challenged udder tissue of lactating cows are shown in Fig. 2. Comparing the RT-qPCR results of RNA depleted and non-depleted samples, we see the highest RNA depletion effect in variant B.

Exemplarily, test RNA-Seq libraries were prepared from depleted and non-depleted RNA samples from the same tissue sample and subjected to paired-end RNA-Seq. Monitoring of the RNase H-mediated depletion effect by RNA-Seq analysis revealed a decreased proportion of reads mapping to the targeted milk protein genes, declining from about 60% in the transcriptome for the non-depleted sample to 30% in the RNA depleted sample in non-challenged tissue samples (data not shown). Thus, the results of the transcriptome analysis essentially confirmed those obtained by RT-qPCR indicating a substantial reduction of the prevalence of milk protein genes in the transcriptome of the mammary gland from lactating cows. Nevertheless, we were still able to detect milk protein gene transcripts by both RT-qPCR and RNA-Seq.

**Fig. 3:**
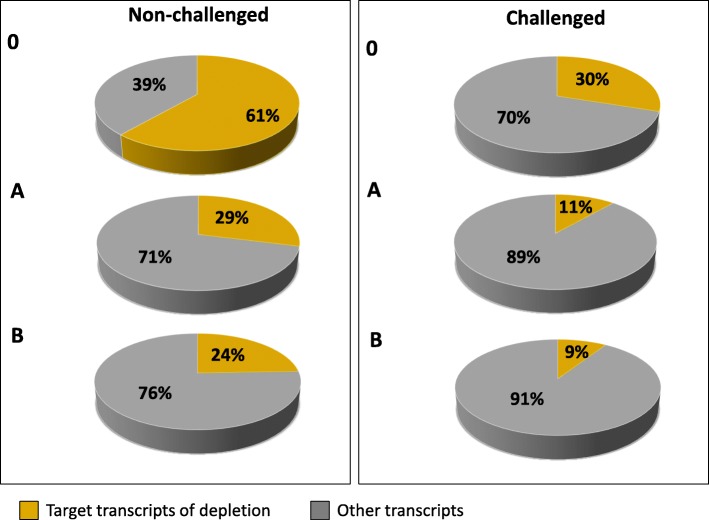
Average proportion of targeted milk protein transcripts in the mammary gland transcriptome with and without depletion in challenged and non-challenged udder samples of three cows, Without RNA depletion (0), with RNA depletion by variant A (A) and B (B). Variant A and B differ in antisense oligonucleotide input for depletion (see Materials and Methods)

However, the use of an RNA depletion approach, which reduces the total milk protein gene content by about 50% in total, enables sequencing costs to be reduced. We can assume that usually about 60% of the genes expressed in the transcriptome of the lactating mammary gland are milk protein genes (see Fig. 3). For describing the complex transcriptome of this tissue, we need at least 30 mill paired-end non-milk protein gene reads and respectively a sequencing depth of about 75 mill raw reads in non-depleted RNA samples. If we can achieve a 50% reduction of the proportion of milk protein transcripts by applying the selective RNA depletion step (i.e. their proportion in the transcriptome is only 30%), a sequencing depth of 42 mill reads would be sufficient for RNA-depleted samples. This would reduce sequencing costs by 50% per sample (currently 625 to 350 € given standard full cost calculation) at additional costs of 8 € per depletion assay. Thus, for break-even, the costs for sequencing 30 million paired-end non-milk protein gene reads would have to be in the dimension of less than about 50 €. A further advantage of the milk protein depletion is savings in computing time and data storage due to lower number of reads for processing.

### Application of the RNaseH-mediated RNA depletion approach in mammary transcriptome sequencing

In a proof-of-principle study on RNA samples from pathogen-challenged and non-challenged udder tissue of lactating cows, transcriptome sequencing analysis has been carried out with a total of 18 RNA-Seq libraries prepared from the mammary gland of three lactating cows. One udder quarter of each cow had been experimentally challenged with *E. coli*. Another udder quarter of the same cow served as a non-challenged control.

RNase H-mediated RNA depletion targeting highly abundant milk protein gene transcripts was carried out on total RNA isolated from challenged and non-challenged mammary gland samples prior to library preparation. RIN values of the non-depleted RNA samples were in a range from 7.7 to 9.6; the scores have declined by about 2 units after RNA depletion treatment, as we had also seen in initial investigations, which may indicate partial RNA degradation. However, the RIN values were not associated to the RNA depletion differences for individual target genes (see below).

Challenge of udder quarters with the pathogen *E. coli* was performed to compare the effects of the targeted milk protein gene depletion procedure in case of lower milk protein synthesis/milk production and to evaluate a possible bias in the samples caused by applying the RNA depletion method.

### Statistics of mammary gland transcriptome sequencing

After adapter trimming of raw reads, 62,510,886 to 92,966,426 reads with a length of 100 bp were obtained for the different mammary gland transcriptome libraries. After read quality trimming, there were between 54,303,133 and 82,880,381 reads available for subsequent transcriptome analysis of depleted and non-depleted RNA samples from challenged and non-challenged udder quarters (Table 1). Guided alignment of these reads to the *Bos taurus* reference genome revealed an average mapping rate of 98% across all 18 samples (Table 1).

**Table 1:** Read statistic of raw data, after quality trimming and read alignment to the bovine genome

Udder quarter treatment	Animal No.	Sample treatment^a^	*RIN* *value*	*Reads (total number)*	*Reads after quality trimming (%)*	*Mapped reads (%)*
Non-challenged	Cow1	0	7.7	77,920,096	90	98
		A	4.9	70,975,008	77	97
		B	4.6	81,396,012	91	98
	Cow2	0	7.8	78,859,264	90	98
		A	4.9	80,349,800	90	98
		B	5.4	78,824,666	89	98
	Cow3	0	8.1	80,203,836	90	99
		A	5.1	62,510,886	89	98
		B	5.6	74,101,514	89	98
Challenged	Cow1	0	7.9	78,564,210	90	98
		A	4.7	78,719,102	90	98
		B	4.9	80,990,200	89	98
	Cow2	0	9.6	92,966,426	89	98
		A	5.9	63,610,608	88	95
		B	6.9	70,529,798	89	97
	Cow3	0	7.8	85,090282	89	98
		A	6.0	81,278,402	89	98
		B	5.7	88,169,812	89	98

^**a**^Pathogen challenged and non-challenged udder tissue samples of the cows 1–3, each of them without RNA depletion (0) and with RNA depletion variant A (A) or variant B (B). Variant A and B differ in antisense oligonucleotide input for depletion (see Materials and Methods)

### Targeted reduction of highly abundant transcripts in mammary transcriptome libraries

Previous transcriptome studies [10, 22, 23] showed that the majority of transcripts expressed in the lactating mammary gland transcriptome mapped to highly abundant milk protein genes. Ibeagha-Awemu et al. [21] have identified the 24 most frequently expressed genes in the bovine mammary gland transcriptome, with the milk protein genes (*LALBA*, *PAEP*, *CSN1S1*, *CSN1S2*, *CSN2* and *CSN3*) accounting for a total of 77% of reads. The study by Cánovas et al. [10] has shown that the milk protein genes targeted by our RNA depletion method are among the six genes most highly expressed in the mammary gland tissue of lactating Jersey and Normande cows. In the transcriptome of ovine lactating mammary gland tissue, 53% of reads covered transcripts coding for milk protein genes [22].

In our study, RNA-Seq analysis of non-depleted RNA samples from non-challenged udder quarters assigned 61% (52–71%) of the total number of mapped fragments to milk protein genes, whereas in *E. coli* challenged udder quarters 30% (0.6–57%) of fragments mapped to transcripts coding for milk proteins (Fig. 3). Analogous to this high variability, a high clinical variability between cows in response to *E. coli* challenge had also been reported in other studies [24, 25] and in our own dataset (see below).

The lower milk protein gene expression levels in challenged mammary samples might be due to an activation of processes required for immune defense in the mammary gland, which has a higher priority than milk protein gene synthesis under challenge conditions, finally resulting in lower milk yield in pathogen challenged cows [26]. Intramammary challenge with *E. coli* caused massive tissue damage as observed during sample collection and in turn led to a decrease in milk production in the cows investigated. Mastitis-associated reduction of milk production and decrease of the milk protein gene expression levels following infection with *E. coli* have been reported in other studies [27–30]. In our experiment, an analogous reduction of milk protein gene expression was observed in the non-depleted (0) and the depleted samples (experimental variants A and B) in *E. coli*-challenges compared to non-challenged samples (Fig. 3).

After selective RNA depletion only 29% (25–36%) (experimental variant A) or 24% (21–30%) (experimental variant B) of the fragments from the non-challenged udder quarters were assigned to the milk protein genes (Fig. 3). The effect of the targeted RNA depletion could also be clearly observed in the challenged udder quarters; only 11% (0–24%) or 9% (0–19%) (variant A or B, respectively) of all fragments mapped to milk protein transcripts.

Altogether, the RNA-Seq analysis of the targeted depletion of milk protein gene transcripts in the RNA pool from lactating mammary tissue showed that the proportion of highly abundant milk protein gene transcripts could be successfully reduced. The RNA depletion effect was observed with both experimental depletion conditions (A and B), independently of the challenge status of the udder tissue and resulted in a reduction of milk protein transcripts by more than 50% compared to the non-depleted sample.

Across all samples, we found that the *CSN2* transcripts were the most abundant milk protein gene transcripts (followed by *CSN1S1*, *CSN3* and *PAEP* transcripts, see Table 2) in the mammary gland of lactating cows, which confirms the results of previous transcriptome profiling reports [10].

**Table 2:** Percentage of fragments mapped to individual milk protein transcripts in relation to the total number of fragments in each sample as counted by featureCounts [50]

Udder quarter treatment	Animal No.	Sample treatment^a^	*LALBA*	*CSN1S1*	*CSN3*	*CSN2*	*CSN1S2*	*PAEP*
Non-challenged	Cow1	0	2.7	15.7	11.5	31.1	4.2	5.5
		A	3.1	17.9	0.6	9.6	0.3	4.5
		B	2.6	12.2	0.2	8.7	0.2	5.9
	Cow2	0	0.8	18.7	9.6	14.3	4.9	3.9
		A	0.5	18.7	0.8	3.1	0.1	2.2
		B	0.4	16.4	0.2	2.8	0.1	2.0
	Cow3	0	1.0	16.7	9.5	21.6	4.9	7.4
		A	1.0	14.3	0.7	4.2	0.1	5.2
		B	0.8	11.6	0.1	3.8	0.1	5.0
Challenged	Cow1	0	1.1	15.2	11.1	21.9	3.3	4.3
		A	1.0	12.1	0.6	5.9	0.1	3.8
		B	0.9	9.0	0.2	5.4	0.0	4.0
	Cow2	0	0.4	12.3	7.5	6.3	2.1	4.2
		A	0.2	6.2	0.2	2.2	0.0	1.8
		B	0.2	4.5	0.0	1.4	0.0	1.7
	Cow3	0	0.0	0.1	0.5	0.1	0.0	0.0
		A	0.0	0.1	0.0	0.0	0.0	0.0
		B	0.0	0.0	0.1	0.0	0.0	0.0

^a^Udder tissue samples without RNA depletion (0), with RNA depletion by variant A (A) or variant B (B). Variant A and B differ in antisense oligonucleotide input for depletion (see Materials and Methods)

However, focusing on individual targeted milk protein genes, we observed some variability of the RNA depletion efficacy. A nearly complete reduction of transcript levels was recorded for *CSN3* and *CSN1S2* (up to 100% of fragments) in depleted samples compared to non-depleted samples (Table 2). A 75% depletion effect was achieved for *CSN2* transcripts with a reduction from an average proportion of 16% of the counted fragments in non-depleted samples to 4% (variant A and B) after RNA depletion. Transcript depletion of *CSN1S1*, *PAEP* and *LALBA* was less comprehensive (Table 2). This indicates that not all these transcripts have been completely removed from the transcriptome after performing the selective RNA depletion procedure.

The different efficiency in the RNA depletion of the individual milk protein transcripts may have different causes, e.g. the formation of secondary or tertiary structures of the targeted milk protein transcript sequences, which may impede the hybridization of the selected antisense oligonucleotides to the respective sequence region. Some possible reasons, however, for the variable efficiency of the RNA depletion across the targeted milk protein genes can be excluded:

Visual inspection of targeted milk protein gene sequences revealed that there are no internal polyA stretches with a number of consecutive A nucleotides > 7 present, which could have compromised the subsequent polyA selection step. Differences in the distance between the selected oligonucleotide position and the 3′ end of the target gene can also be excluded as a possible general cause for variable RNA depletion efficiency, since the *CSN2* gene was very successfully depleted, although its capture oligonucleotides are located at a distance of 284 and 439 nucleotides from the 3′ end of the gene. Across all milk protein genes, the selected oligonucleotides had a medium distance of about 280 bp to the 3′ end of the target gene (range from 35 to 443). However, it is conceivable that it would be more advantageous to select the capture oligonucleotides as close as possible to the 3′ end of the gene to be depleted, which was not always possible due to the inherent sequence properties of individual genes (e.g., repetitive elements, known sequence variation).

Another reason for insufficient depletion of target transcripts might be genetic variants localized in the gene section covered by the oligonucleotide used for RNA depletion. However, transcript sequence analysis of the individuals included in this study revealed that SNPs in capture oligonucleotide sequences could only be detected for *CSN3*. As *CSN3* transcripts were almost completely depleted, obviously this sequence variation did not affect the depletion efficiency.

However, the RNA depletion approach may have limitations, if the animals to be analyzed show not yet detected alternative splice sites in the targeted gene. The visual inspection of read alignments using the Integrative Genomics Viewer (IGV) revealed that the lower RNA depletion efficiency observed for *CSN1S1* can be due to the existence of a high number of alternative splice variants, which were not all addressed by the capture oligonucleotides designed. The new bovine genome annotation ARS-UCD1.2 at NCBI that has been published very recently displays 25 transcript variants predicted based on now available RNA-Seq data (https://www.ncbi.nlm.nih.gov/gene/282208), not all of which were known at the time of primer design. Both *CSN1S1* target oligonucleotides are located in different splice sites, where an intron may be inserted, so that some highly expressed transcript variants cannot be covered. In this case, adding additional capture oligonucleotides in the RNA depletion assay, which cover all *CSN1S1* transcript variants may be beneficial in future iterations of the depletion protocol. For the other genes with incomplete RNA depletion efficiency, *PAEP* and *LALBA,* neither alternative splice variants nor genetic variation in oligonucleotide sequences were detected.

**Fig. 4:**
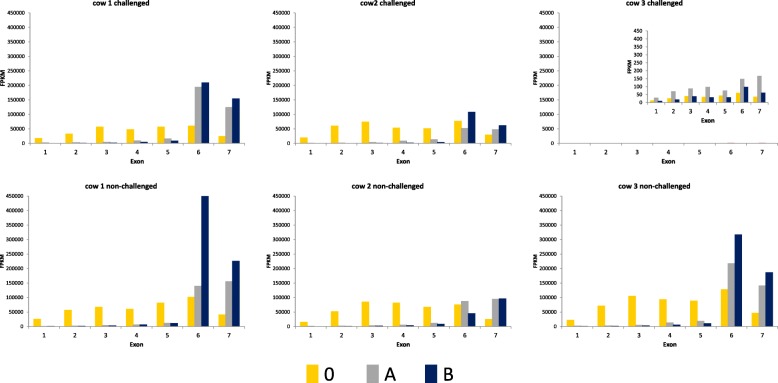
Fragment abundance (in FPKM) per exon of the *PAEP* gene for depleted (protocol variants A and B) and non-depleted (0) RNA samples from *E. coli* challenged and non-challenged udder quarters for all cows included in the challenge experiment. For cow 3 challenged, almost no milk protein gene expression was observed: see y axis scale of the inserted diagram

The visual inspection of *PAEP* and *LALBA* read alignments using the IGV showed that their reads in depleted RNA samples were biased toward increased coverage at the 3′ end of the respective gene. In these samples, a clear decline of read coverage is visible behind the last nucleotide of the capture oligonucleotide in 5′ direction of the gene as expected. Respective non-depleted samples showed no analogous decline in read coverage, (see Fig. 4, Additional file 1). These data indicate that the observed trend of a positional bias towards an increased coverage at the 3′ end of the gene may be associated with some limitations in the RNA depletion procedure. Obviously, the 3′ fragments of transcripts, which carry the polyA-site and remain in the RNA pool after RNase H degradation, are still captured in the poly A+ selection step of the library preparation and finally compromise the depletion of the corresponding genes. Our data indicate that the depletion efficiency of *PAEP* and *LALBA* could possibly be improved by selecting the capture oligonucleotides as close as possible to the 3′ end of the gene.

### RNA depletion maintains individual mammary gland milk protein gene expression patterns in cows challenged with the pathogen *E. coli*

As already mentioned above (Fig. 3), the proportion of milk protein transcripts in the mammary gland transcriptome is lower after intramammary challenge with *E. coli* compared to the non-challenged control tissue. This can probably be explained by severe tissue damage resulting in cell destruction followed by a reduced milk protein synthesis and can also be due to an active defense against the pathogen with associated upregulation of immune defense genes. Monitoring of the expression profiles of selected transcripts coding for milk protein transcripts in each cow showed individual changes after pathogen challenge (Table 2). In all three cows, reduced expression of milk protein transcripts in non-depleted and depleted samples was evident after *E. coli* challenge, whereby cow 3 showed an almost complete lack of target gene expression after intramammary challenge. This indicates that the milk protein synthesis in the mammary gland tissue of the challenged udder quarter of this cow had almost completely stopped. Indeed, with only about 38% of the original amount of milk in the challenged udder quarter, this cow showed the largest drop in milk yield after *E. coli* challenge compared to the other two cows enrolled in this experiment. In contrast to the other two lactating cows, the pathological report of cow 3 showed granulocyte infiltration into the tissue of the challenged udder quarter. A need for RNA depletion would not have been required for this sample per se, which is clearly reflected in the results obtained. However, to avoid experimentally induced bias when comparing challenged and non-challenged udder quarters in a challenge experiment, identical treatment of both samples is essential.

### Effect of RNA depletion targeting highly abundant transcripts on the detection of other transcripts in the transcriptome

**Fig. 5:**
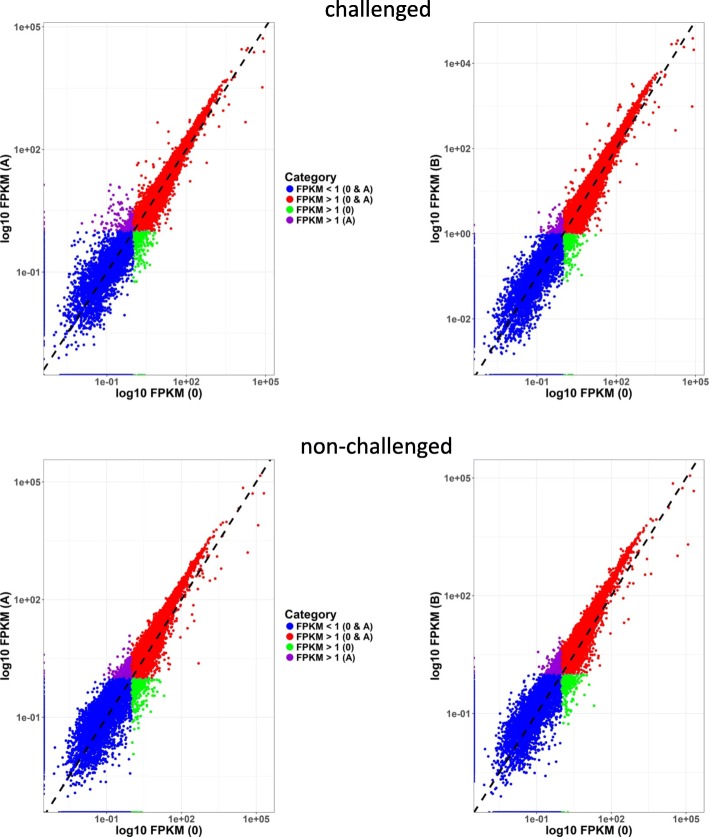
Correlation plot of mean gene expression level (in log10 FPKM) between depleted and non-depleted RNA samples within *E. coli* challenged and non-challenged udder samples across animals investigated. 0, A, and B represent the non-depleted or the depleted RNA samples (protocol variant A and B), respectively

**Fig. 6:**
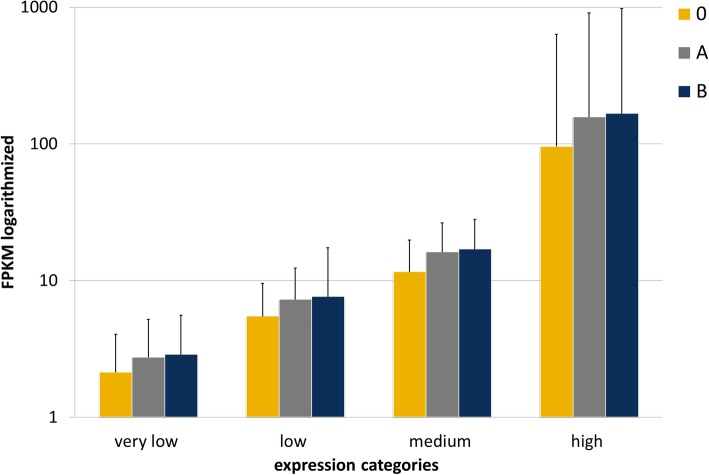
Effect of RNA depletion on transcriptome expression levels (mean and standard deviation within expression categories). Expression categories are classified in quartiles according to the transcription level based on average FPKM values across samples from challenged and non-challenged udder quarters. Sample treatment: no RNA depletion (0), RNA depletion variant A (A) or RNA depletion variant B (B). Variants A and B differ in antisense oligonucleotide input for RNA depletion (see Materials and Methods)

A correlation analysis between FPKM data for depleted and non-depleted RNAs from the same udder quarter demonstrated that the selective RNA depletion procedure did not introduce a systematic bias into gene quantification (see Fig. 5, Additional file 2). The density plots for monitoring the distribution of quantitative gene expression revealed a shift towards higher levels for the variants A and B compared to the non-depleted samples (see Additional file 3). To evaluate the targeted RNase H-mediated RNA depletion effect on the sensitivity of RNA-Seq analysis, expression quartiles of the transcripts were formed, graded according to their transcription level. Monitoring of the RNA depletion effect on the average FPKM value of the different expression categories revealed that they shifted to higher values in consequence of RNA depletion indicating an improved sensitivity of RNA-Seq analysis (Fig. 6). The average FPKM of the “very low” transcript expression quartile has increased from 2.1 in the non-depleted samples to 2.7 (variant A) and 2.9 (variant B) after RNA depletion. In the “low expression” quartile, the average FPKM value has risen from 5.5 to 7.2 and 7.6 in variant A and B, respectively. The FPKM values of transcripts of the “medium expression” quartile increased from 11.6 to 16.1 and 16.9, and in the “high expression” transcript quartile from 95.6 to 156.8 and 166.5, in variant A and B, respectively. The two methodological RNA depletion variants did not differ substantially (Fig. 6).

In addition, we separately explored the number of genes with an FPKM > 1 that exceeded this threshold only in depleted or only in non-depleted RNA samples. For these comparisons, we analyzed the RNA-Seq read count data based on the Ensembl 87 reference annotation. Table 3 indicates that we had a net surplus of 239 or 278 genes for the depletion groups A and B, respectively. Additional file 4 illustrates the overlap in genes with FPKM > 1 in RNA depleted and non-depleted samples.

**Table 3:** Number of expressed genes with FPKM > 1 and of genes with changed FPKM values after selective RNA depletion for all groups based on the Ensembl 87 reference annotation

Groups^a^	All genes with FPKM > 1	Genes with FPKM > 1 only after RNA depletion	Genes with FPKM > 1 before and FPKM < 1 after RNA depletion
0	12,546	/	/
A	12,773	554	315
B	12,817	582	304

^a^Samples without RNA depletion (0) and with RNA depletion according to variants A (A) and B (B). Variants A and B differ in antisense oligonucleotide input for RNA depletion (see Materials and Methods)

### Differential expression analysis of loci in response to intramammary challenge

To evaluate whether the depletion of highly abundant transcripts causes a bias in physiological reaction patterns in response to an *E. coli* challenge and whether the detection of significantly differentially expressed transcripts can be improved, a differential expression analysis of *E. coli* challenge vs. control was performed.

**Fig. 7:**
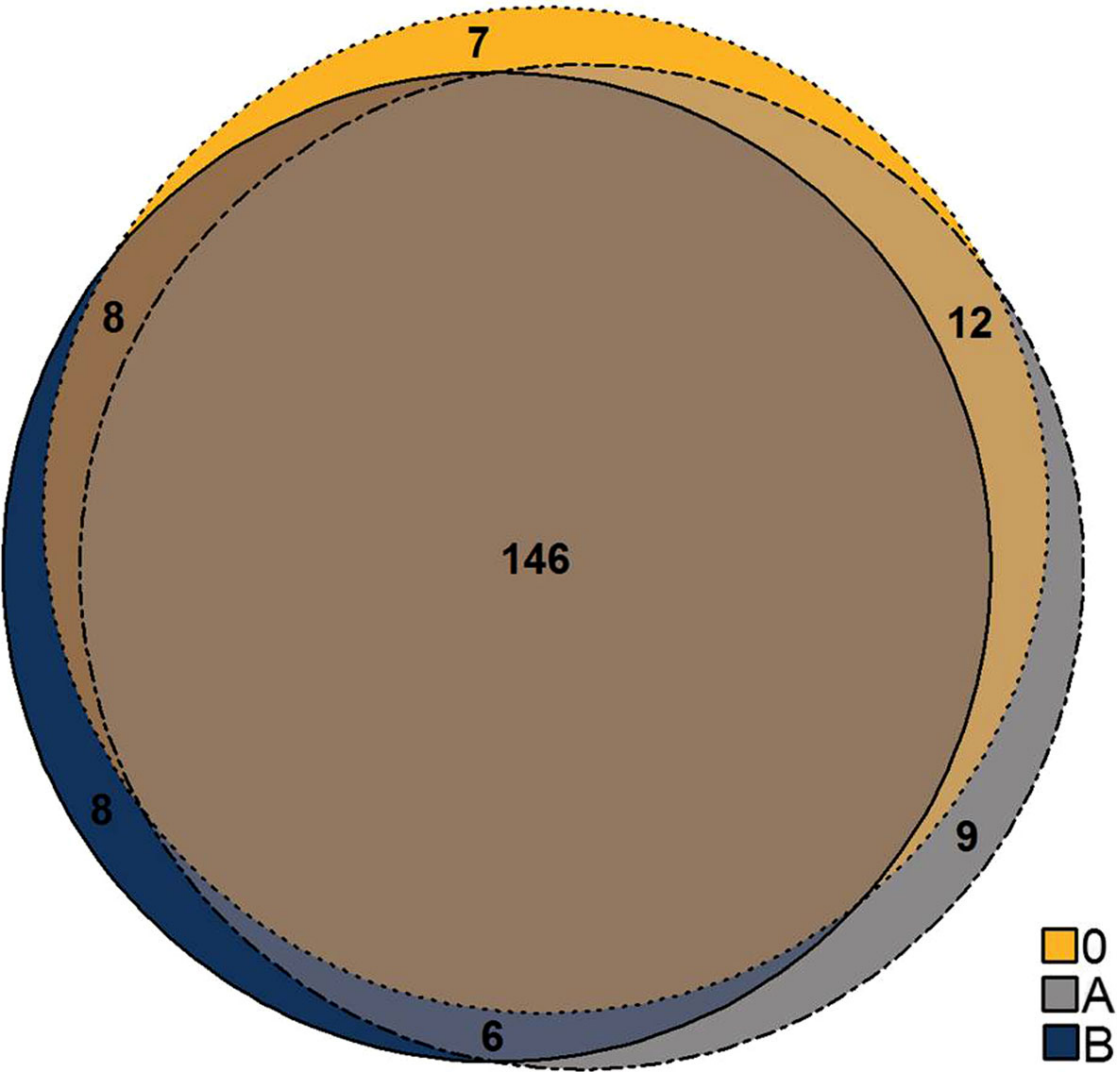
Number of pathways enriched for significantly differentially expressed genes after *E. coli* challenge in depleted (A and B) and non-depleted (0) udder tissue samples. Variant A and B differ in antisense oligonucleotide input for RNA depletion (see Materials and Methods)

Non-depleted samples (0) showed 1079 significantly differentially expressed loci (for list of loci see Additional file 5). Targeted RNA depletion of highly abundant milk protein transcripts resulted in 1290 (variant A) and 1226 (variant B) significantly differentially expressed loci (for the list of loci see Additional file 5), which demonstrates a clear increase in number when applying the RNA depletion method. Overall, the transcriptomic determination of general physiological reaction patterns after *E. coli* udder challenge should not be compromised by the RNA depletion procedure. To monitor this, significantly differentially expressed biological pathways in challenged and non-challenged samples were analyzed based on differentially expressed loci. Ingenuity pathway analysis (for lists of significantly enriched canonical pathways see Additional file 6) showed high agreement between the groups (0, A and B) with 146 identified common biological pathways, which were consistently enriched in the transcriptome of both non-depleted (0) and depleted samples (A and B) after *E. coli* challenge (Fig. 7). The number of pathways that only occurred in one or two of the groups was low (6–12 pathways). This indicates that the RNA depletion procedure does not result in an unintentional bias in the global biological signaling pathway patterns in response to *E. coli* challenge.

Further, we tested for potential differential expression of individual genes well described in literature to be affected by *E. coli* challenge in the mammary gland [31]. We had a specific focus on immune response processes and looked particularly at genes encoding cytokines as well as chemokine and inflammatory relevant receptors. The comparison of the transcription profiles in response to the *E. coli* challenge between the methodological groups (0, A and B) showed that RNA depletion improved the sensitivity to detect such well-known effects on gene expression. We observed that significantly differential expression of some cytokine encoding genes in response to *E. coli* challenge was only detectable in the depleted samples A and B in contrast to the non-depleted samples.

**Fig. 8:**
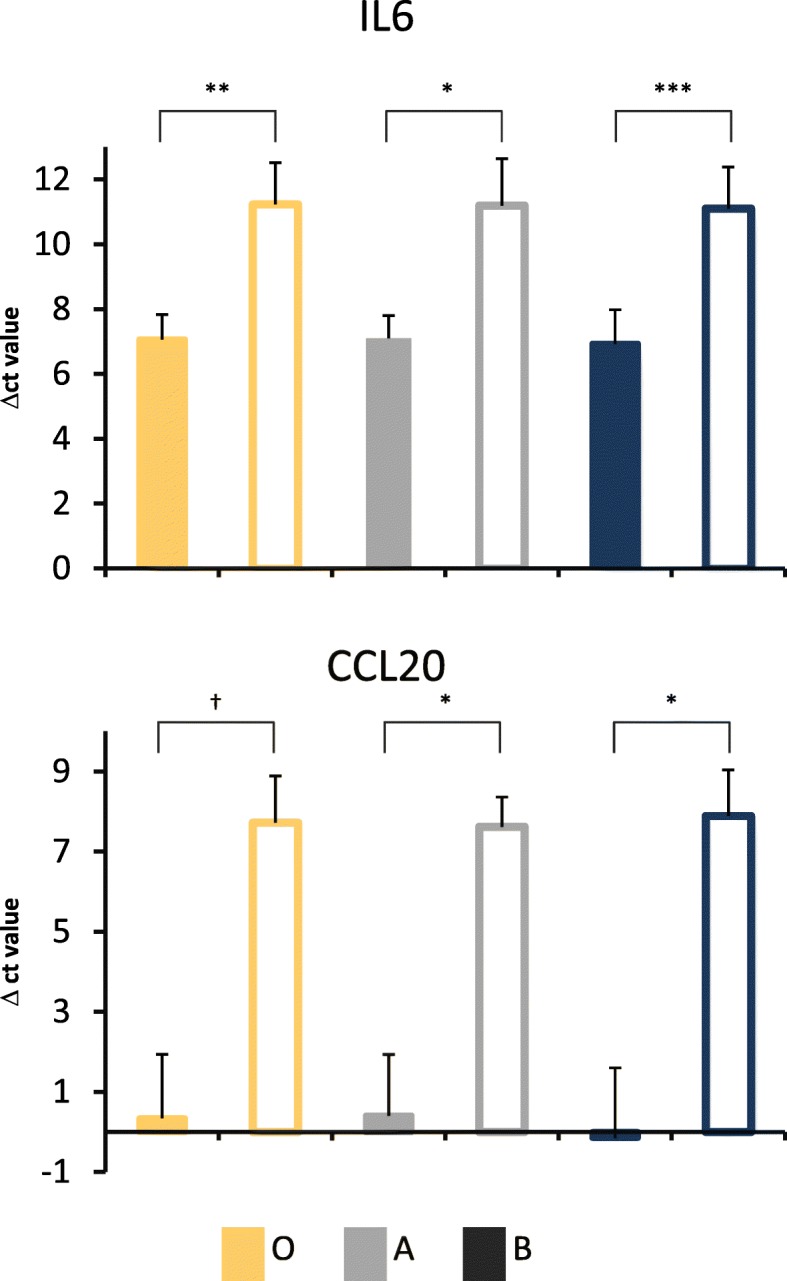
Differential expression analysis testing immune genes for response to pathogen challenge. Results from RT-qPCR for the *IL6* and *CCL20* genes in response to *E. coli* challenge: samples from challenged (filled boxed) and non-challenged (open boxes) udder samples of three cows. Variants A and B differ in antisense oligonucleotide input for RNA depletion (see Materials and Methods), while 0 comprises the non-depleted RNA. *: *p* < 0.05, **: *p* < 0.01, ***: *p* < 0.001, †: *p* < 0.10

This included the master regulator of acute phase response *IL6* gene [32, 33] (Table 4) and the *CCL16* gene. The *CCL20* gene was found to be significantly differentially expressed only in samples processed with RNA depletion variant B. These cytokines and chemokines are known to be regulated after challenge with *E. coli* [34–36]. For relevant transmembrane receptors, we observed a challenge-related, significantly different expression only in depleted samples. This applied to *TREM1* (triggering receptor expressed on myeloid cells 1) gene, a critical regulator of diverse cellular functions including amplification of inflammation [37], and the *CCR2* and *CCR7* genes, both encoding G-protein coupled receptors, which are essential for leukocyte recruitment [36]. A targeted differential RT-qPCR analysis for the *IL6* and *CCL20* genes between pathogen-challenged and non-challenged tissue samples confirmed a higher sensitivity for detecting a response to pathogens in depletion variant B (Fig. 8).

**Table 4:** Differential expression obtained from RNAseq data for selected genes in non-challenged udder samples compared to *E. coli* challenged samples

Gene	q-value^1^		
	0^2^	A^2^	B^2^
*IL6*	0.092	0.019	0.002
*CCL20*	0.662	0.289	0.002
*CCL16*	0.057	0.019	0.004
*TREM1*	0.096	0.036	0.002
*CCR2*	0.189	0.200	0.022
*CCR7*	0.124	0.051	0.024

^1^ Statistical significance (q value) for differential expression between infected and non-infected samples

^2^Udder tissue samples without RNA depletion (0), with RNA depletion according to variants A (A) and B (B). Variants A and B differ in antisense oligonucleotide input for depletion (see Materials and Methods)

The highly abundant milk protein transcripts obviously seem to impede the detection of these exemplarily selected, lowly expressed transcripts in the analysis of transcriptomes using RNA-Seq. Thus, the depletion of the milk protein transcripts from the total RNA pool prior to RNA-Seq offers a higher sensitivity for detecting relevant biological signaling processes in global transcriptome analysis of mammary gland tissue from lactating cows, and thus enables or improves the detection of genes expressed at a lower level.

### Detection of unknown genes

By improving the sensitivity of a RNA-Seq analysis due to selective depletion of highly abundant transcripts, it is expected that also an improvement in detection of yet unknown transcripts will be achieved. After generating transcriptome annotations separately for the non-depleted and depleted experimental group (0, A, B, without considering the challenge status), the total number of expressed loci found in the transcriptomes of each group was determined. Non-depleted samples showed a lower number of expressed loci than samples depleted with variant A or B (Table 5). Even after applying an expression threshold value of FPKM > 1, a clearly higher number of loci were detected in depleted samples than in non-depleted samples. A similar result was obtained when analyzing for yet unknown loci. For RNA-depleted samples, a higher number of these loci yet unannotated in the *Bos taurus* Ensembl annotation were identified compared to non-depleted samples, also at a threshold value of FPKM > 1. The application of our RNA-depletion approach thus provides a generally higher number of discovered loci and facilitates the detection and analysis of new loci.

**Table 5:** Number of all expressed loci and the subset of unknown expressed loci based on a group-specific transcriptome annotation

Groups^a^	All loci	All loci with FPKM > 1	Unknown loci	Unknown loci with FPKM > 1
0	32,434	16,168	4411	1915
A	43,331	19,396	11,023	2997
B	35,510	17,779	6343	2509

^a^Samples without RNA depletion (0) and with RNA depletion according to variants A (A) and B (B). Variants A and B differ in antisense oligonucleotide input for depletion (see Materials and Methods)

## Conclusions

The results of our study suggest that the sensitivity of the RNA-Seq analysis is improved when removing highly abundant milk protein gene transcripts from the total RNA pool of the mammary gland from lactating cows prior to RNA-Seq library preparation (negative selection). The implementation of a selective RNase H-mediated RNA depletion of milk protein gene transcripts from the transcriptome of the mammary gland tissue of lactating cows will help to achieve a more comprehensive transcript catalogue of the mammary gland transcriptome, which better reflects its complexity. At the same time, our data confirm that targeted milk protein transcript depletion does not introduce a bias in the outcome of transcriptome analyses within mammary gland pathogen challenge experiments.

Depletion variant B is superior to A for a number of parameters: stronger depletion of milk protein transcripts (Fig. 3, Table 2), higher number of genes (from the reference annotation Ensembl 87) with FPKM > 1 not found in non-depleted samples (Table 3), higher number of differentially expressed genes found in *E. coli* challenge (Additional file 5) and small, but consistently higher FPKM values for gene expression quantiles (from the reference annotation Ensembl 87, Fig. 6). This suggests that a higher and equimolar concentration of the antisense oligonucleotides in RNA depletion assay should be applied in follow-up studies.

## Methods

### Animals and samples

Twelve Holstein Friesian cows were challenged during their first lactation with *E. coli* for 24 h [38]. The animals were purchased from ordinary commercial dairy farms in the northeast region of Germany. One udder quarter of each cow was challenged with 500 colony forming units of *E. coli 1303*. The intramammary challenge was performed at day 36 ± 3.4 after first parturition, 24 h before dissection of the cow. One of the remaining untreated udder quarters was used as control. The cows were separately housed in a loose-stall barn and milked twice daily. Environmental conditions and feeding during the calving period and the challenge interval were identical for all cows enrolled in the study as previously described [38]. Of these challenged 12 animals, three lactating cows without clinical signs of diseases at the start of the intramammary challenge, but with different degrees of clinical response to pathogen challenge were used to validate the RNase H-mediated RNA depletion method targeting milk protein genes highly expressed in the mammary gland. Two of the cows included in this experiment (cow 1 and 3) are offspring from the same sire, but the maternal ancestry is different for all cows. Mammary gland parenchymal tissue of each quarter from these three cows was collected immediately after killing and dissection, frozen in liquid nitrogen and stored at − 80 °C.

### RNA preparation

For each of the three cows, frozen samples (50 mg) of parenchymal tissue from the challenged and from a control (non-challenged) udder quarter were pulverized in liquid nitrogen, and total RNA was extracted with TRIzol reagent (Invitrogen, Darmstadt, Germany) followed by an on-column-purification using the NucleoSpin RNA II kit (Macherey & Nagel, Düren, Germany) with modifications of the DNase digestion step according to Weikard et al. [39]. After testing the total RNA preparation for genomic DNA presence by PCR [40], the DNase-treatment step was repeated when necessary. The RNA concentration was measured with a Qubit Fluorometer (Invitrogen, Germany). For RNA quality control, RNA integrity was determined using the 2100 Bioanalyzer Instrument (Agilent Technologies, Germany).

### Depletion of highly abundant transcripts

The depletion procedure of highly abundant transcripts originating from the casein gene cluster (*CSN1S1*, *CSN1S2, CSN2* and *CSN3*), α lactalbumin gene (*LALBA*) and progestagen associated endometrial protein gene (*PAEP*) in lactating mammary gland tissue is based on hybridization of antisense oligonucleotides specific for these target transcripts to total RNA prior to library preparation used for RNA-sequencing (RNA-Seq). The targeted RNA depletion procedure is presented in Fig. 1. Two antisense oligonucleotides with a melting temperature > 65 °C were derived of each target gene reference sequence, *Bos taurus* reference genome assembly UMD3.1 (see Table 6) using the OLIGO Primer Analysis Software (MedProbe, Oslo, Norway). For the selection of antisense oligonucleotides that are specific for the targeted milk protein genes, care was taken that they are located as far as possible in exons located close to the 3′ end of the gene to be depleted and outside of repetitive and low complexity sequence regions as well as known genetic variants and alternative splice sites of the targeted genes. To avoid off-target amplification the specificity of the selected oligonucleotides (Table 6) was checked by BLAST search against the *Bos taurus* reference transcriptome and genome assembly (UMD3.1, annotation release 105 [41]) using the Primer-BLAST tool [42].

**Table 6:** Antisense oligonucleotide sequences designed for targeting highly abundant transcripts in the mammary gland

Gene name	Accession number	Oligonucleotide name	Oligonucleotide sequence (5′-3′)
*PAEP*	NM_173929.3	LGB_R5	GGGCTCACCTAGATGTGGCACTGCTC
		LGB_R6	GCTCAGCACTGTTCTCCATGCAGAAG
*LALBA*	NM_174378.2	LALBA_R6	GTCATCAGTAAGATCATCATCCAGGAAC
		LALBA_R7	CAGAACAGAGTGCTTTATGGGCCAACC
*CSN1S2*	NM_174528.2	CSN1S2_R3	ATAACCAGGTAGAAGCAGTTAATTCCAG
		CSN1S2_R4	ATGCTGGTTGTATGAAGTAAAGTGGTAG
*CSN1S1*	NM_181029.2	CSN1S1_R7	TCAGAATTCACTTGACTCCTCACCACAG
		CSN1S1_R8	TAGGATTAGGGATGTCAGAGAATGATGG
*CSN3*	NM_174294.2	CSN3_R6	GTAAGAGGAGACGAGGAAGGAGCCAG
		CSN3_R7	ACTGTGTTGATCTCAGGTGGGCTCTC
*CSN2*	XM_010806178.1	CSN2_R4	TCCAGTCGCAGTCAATTCAAAAGTGAG
		CSN2_R5	CTTTCTGGGGAACAGGCAGGACTTTG

The effect of concentrations of the antisense oligonucleotides in the hybridization assay was tested in two different experimental conditions (Table 7). In the first variant (A), the final oligonucleotide concentration was adjusted according to the expression level of the respective target gene in lactating mammary gland known from other studies [10]. In the second variant (B), a constant final equimolar concentration of 25 μM was applied for each oligonucleotide in the hybridization assay (Table 7).

**Table 7:** Final concentration of the antisense oligonucleotides in the RNA depletion assays

Oligonucleotide pair	Concentration [μM]	
	Variant A	Variant B
LGB_R5, LGB_R6	16.66	25.0
LALBA_R6, LALBA_R7	16.66	25.0
CSN1S2_R3, CSN1S2_R4	8.33	25.0
CSN1S1_R7, CSN1S1_R8	16.66	25.0
CSN3_R6, CSN3_R7	9.0	25.0
CSN2_R4, CSN2_R5	25.0	25.0

Duplicates of 1 μg of total RNA from each sample were denatured by incubation for 2 min at 95 °C in hybridization buffer (10 mM Tris-HCl, pH 7.6, 20 mM KCl) and hybridized for 5 min at 65 °C with antisense oligonucleotides pooled in a total volume of 10 μl. The reactions were carried out in a thermocycler and completed with cooling down to 4 °C.

Immediately after hybridization of the RNA samples with the antisense oligonucleotides, a selective digestion of the RNA strand of the RNA-oligonucleotide hybrids was carried out with RNase H (#AM2292, Ambion). The RNase H reaction mixture consisting of 2 U RNase H and 20 U SUPERase-In (inhibiting RNase A, B, C, 1 und T1, Ambion, #AM2694) in RNase H-buffer (20 mM Tris-HCl, pH 7.6, 4 mM MgCl_2_, 0.02 mM DTT) was prepared on ice. The hybridized RNA-oligonucleotide hybrids were added to 10 μl of RNAse H reaction mixture, mixed, incubated at 37 °C for 10 min and cooled down to 4 °C. To stop the reaction, 1 μl 0.5 M EDTA was added immediately. After shortly spinning down the reaction mixtures the respective duplicates of the samples were pooled, and finally the pooled samples were purified with the RNeasy MinElute Cleanup kit (Qiagen). The RNA quality was controlled with the Agilent Bioanalyzer 2100 and the RNA quantification was carried out using the Qubit Fluorometer.

### Monitoring of the RNA depletion effect by RT-qPCR

Depleted and non-depleted RNA samples were reverse transcribed to cDNA using the SuperScript First-Strand Synthesis System III (Invitrogen, Thermo Fisher Scientific) according to the manufacturer’s instructions. RT-qPCR analysis was performed as described (Weikard et al. 2012) on a LightCycler qPCR platform (Roche). Milk protein gene copy numbers were normalized against those of *RPS15A* used as a reference gene. Primers for transcript quantification via qPCR of each target gene were designed based on the respective reference gene sequence, *Bos taurus* reference genome assembly UMD3.1 (see Table 8). Primer pair specificity was checked by BLAST search against the *Bos taurus* reference transcriptome and genome assembly (UMD3.1, annotation release 105 [41]) using the Primer-BLAST tool [42]. Sequences for gene-specific PCR primers are provided in Table 8.

**Table 8:** Sequences of primers used in RT-qPCR

Gene name	Gene accession number	Oligonucleotide name	Oligonucleotide sequence (5′-3′)
*PAEP*	NM_173929.3	LGB_F1	CAAGATCCCTGCGGTGTTCAAG
		LGB_R1	ACTGTTCTCCATGCAGAAGAGC
*LALBA*	NM_174378.2	LALBA_F2	CAGTTTGCCTGAATGGGTCT
		LALBA_R3	GATCATCATCCAGGAACTTGTC
*CSN1S2*	NM_174528.2	CSN1S2_F2	CACCAGTGAGGAAAATTCAAAG
		CSN1S2_R1	GCTGATAAACAGTCTTGAGATAC
*CSN1S1*	NM_181029.2	CSN1S1_F4	GTGCTGAGGAACGACTTCACAG
		CSN1S1_R4A	CAGTGTATTGTGTGCCTAGTGG
*CSN3*	NM_174294.2	CSN3_F5	GCCCACCTGAGATCAACACAG
		CSN3_R5	AGGAGACGAGGAAGGAGCCAG
*CSN2*	XM_010806178.1	CSN2_F1	TGAGGAACAGCAGCAAACAG
		CSN2_R1	ACAGGGGTTTGAGTAAGAGG
*RPS15A*	NM_001037443.1	RPS15A_F1	CCGTGCTCCAAAGTCATCGTC
		RPS15A_R1	TGAAACCAAACTGACGGGATG
*CCL20*	NM_174263.2	CCL20_F3	CAGCAAGTCAGAAGCAAGCAA
		CCL20_R1	CCCACTTCTTCTTTGGATCTGC
*IL6*	NM_173923.2	IL6_F1	GGAGGAAAAGGACGGATGCT
		IL6_R1	GGTCAGTGTTTGTGGCTGGA
*PPP1CC*	NM_174581.2	PPP1CC_F1	TCGACAGCATCATCCAACG
		PPP1CC_R1	CGGGAAACCACCGTACTCA

### Library preparation and RNA sequencing (RNA-Seq)

The effect of the RNA depletion on the transcript composition of the respective samples was monitored by comparative transcriptome analysis of depleted (variant A and B) and non-depleted RNA samples (0), from the challenged and an non-challenged control udder quarter using RNA-Seq. Stranded indexed poly(A+) selected libraries were prepared from 250 ng depleted or non-depleted RNA of mammary gland samples using the TruSeq RNA Library Preparation Kit v2 set A (Illumina). The standard procedures for Illumina’s mRNA-Seq were applied, and the libraries were subjected to paired-end (2 × 100 bp) mRNA sequencing in two lanes on the HiSeq 2500 Sequencing System (Illumina) [43].

### Read mapping

After removal of adapters with Cutadapt (version 1.13) [44], quality control with FastQC [45] and read quality trimming with QualityTrim (version 1.6.0) [46], read mapping was performed with HISAT2 [47] against the bovine reference genome (UMD3.1 [48], Ensembl-Annotation release 87 [49]).

### Evaluation of the RNA depletion success

To assess the success of the targeted RNA depletion of the milk protein gene transcripts, the proportion of these transcripts in the transcriptomes of depleted (variant A and B) and non-depleted control samples of challenged and control udder tissue was determined. FeatureCounts [50] was used to calculate sequencing fragments assigned to the loci present in the *Bos taurus* genome annotation (Ensembl-Annotation release 87). First, all sequencing fragments of a sample were summed up (=100%) using R, version 3.4.3 [51]. Afterwards, only those fragments, which were assigned to the target genes (*CSN1S1, CSN1S2, CSN2, CSN3, LALBA* and *PAEP*) were counted, and the respective fraction across all target genes was calculated for each sample. Finally, averages of all three animals for control and depleted samples were calculated from these values and compared. In addition to average values across all genes in depleted and non-depleted, challenged and non-challenged samples, fragments for single milk protein genes of each udder quarter were analyzed and compared for a more detailed evaluation.

### Analysis of expression categories

To determine if/how the general expression patterns of genes of the mammary transcriptome were affected by the RNA-depletion procedure, the transcripts present in the mammary transcriptome of lactating udder tissue were grouped into expression categories based on their transcript expression levels.

Fragments per kilobase per million fragments mapped (FPKM) were calculated for all loci based on the fragment counts determined by featureCounts. To define the limits of the expressions categories, an average FPKM for each locus across all non-depleted samples (challenged and non-challenged) was calculated. All loci with an average FPKM value lower than 1.0 and the six milk protein genes targeted by RNA depletion were removed from the dataset.

From this final dataset, 25, 50 and 75%- quartiles were calculated. The limits of expression categories were defined accordingly. The expression quartiles comprised transcripts with FPKM values ranging from 1 to 3.55 FPKM (“very low”), 3.55 to 7.75 FPKM (“low”), 7.75 to 17.19 FPKM (“medium”) and 17.19 to 21,068.1 FPKM (“high”).

In the next step, average FPKM values of all transcripts of each defined expression quartile were calculated. This was performed for the non-depleted as well as the depleted samples (variants A and B) separately, but with challenged and non-challenged samples together.

### Analysis of depletion effect on gene expression level by read counts from RNA-Seq

Read counts per gene as obtained by featureCounts from the Subread package [50] based on the reference Ensembl 87 annotation were further screened for a potential bias introduced into gene quantification by correlation analysis between depleted and non-depleted RNA for each udder quarter and also within challenged and non-challenged groups. Furthermore, we also performed an exon-wise expression analysis of all milk protein genes targeted by depletion and inspected the alignment files by Integrated Genomics Viewer, IGV [52].

### Evaluation of the RNA depletion effects on the *E. coli* challenge response

Investigation of differential gene expression in response to *E. coli* challenge and whether this is affected by the targeted RNA depletion of highly abundant transcripts was performed using Cuffdiff (v.2.2.1) [53] with default parameter settings based on the *Bos taurus* UMD 3.1 Ensembl v87 genome annotation. The differential gene expression of challenged versus non-challenged udder tissue samples was compared in both experimental RNA depletion variants (A and B) and the control group separately (0), respectively. Thereafter, using R-scripts [51] the total number of significantly differentially expressed loci (q < 0.05) in response to pathogen challenge exceeding a threshold of FPKM = 1 were counted and compared between technical groups (control, variant A, variant B).

The Cuffdiff output of each group (0, A and B) was also used for biologically functional pathway analyses performed with the Ingenuity Pathway Analysis software (IPA) [54]. IPA analysis was performed on lists of loci identified as significantly differentially expressed (q < 0.05). Results of this analysis are presented in a venn diagram, created with the eulerr package [55] in R [51].

### Targeted RT-qPCR for genes with different expression in response to *E. coli* challenge

Depleted and non-depleted RNA samples were reverse transcribed to cDNA and RT-qPCR analysis was performed essentially as described above. Primer sequences of transcripts subjected to quantification are provided in Table 8. Gene expression levels (cycle quantification, Cq values)*,* were normalized against that of the *PPP1CC* gene serving as reference gene. Differential expression between challenged and non-challenged samples was analyzed by t-tests within non-depleted and RNA-depleted samples.

### Detection of unknown genes

To evaluate the RNA depletion effect on the capability to detect yet unknown loci, we used StringTie [56] to perform a reference-based (UMD3.1 [46], Ensembl annotation release 87 [47]) transcriptome assembly for each group (0, A, B) separately. The mapped reads of each dataset of each animal were assembled and the individual gtf files subsequently merged according to the groups, resulting in three group-specific transcriptome annotations.

Based on these annotations the total number of loci expressed in each group was determined with featureCounts. To this end, an average FPKM value for each locus was calculated. To improve the specificity of the data and remove potential background noise those loci with an FPKM < 1 were removed from the dataset.

In addition, the group-specific assemblies were evaluated with regard to unknown/new loci with GffCompare [57]. The detected transcripts of the output were computed into loci and the number of those loci with the class code “u” (unknown) and consisting of more than one exon were counted and compared between the groups.

## Additional files

Additional file 1: IGV screen shot of the read distribution across the *PAEP* gene for the *E. coli* challenged udder sample from cow 2. O, A and B represent the non-depleted or the depleted RNA samples (protocol variant A and B), respectively. Exon 6 is zoomed in for better demonstration of the position of the antisense capture oligonucleotide (see red line). (PDF 235 kb)

Additional file 2: Correlation plot of gene expression level (in log10 FPKM) between depleted and non-depleted RNA samples within *E. coli* challenged and non-challenged udder samples of each cow investigated. 0, A, and B represent the non-depleted or the depleted RNA samples (protocol variant A and B), respectively. (PDF 1551 kb)

Additional file 3: Distribution of gene expression level (FPKM) for the depleted and non-depleted RNA samples from non-challenged udder samples. 0, A, and B represent the non-depleted or the depleted RNA samples (protocol variant A and B), respectively. (PDF 283 kb)

Additional file 4: Number and overlap of expressed genes with FPKM > 1 in depleted (protocol variants A and B) and non-depleted (0) RNA samples. (JPG 645 kb)

Additional file 5: Lists of differentially expressed genes comparing pathogen-challenged and non-challenged udder quarters for non-depleted (0) and depleted RNA samples (protocol variants A and B) at q < 0.05 used for upload into IPA pathway analysis. (XLSX 70 kb)

Additional file 6: Lists of IPA canonical pathways significantly enriched for differentially expressed genes in non-depleted (0) and depleted RNA samples (protocol variants A and B) from pathogen-challenged and non-challenged udder quarters. Pathways are classified for being observed across all protocols (0, A or B) or shared between two or all three protocols. (PDF 214 kb)

## Abbreviations

CCL16: C-C motif chemokine ligand 16
CCL20: C-C motif chemokine ligand 20
CCR2: C-C motif chemokine receptor 2
CCR7: C-C motif chemokine receptor 7
CSN1S1: Casein α S1
CSN1S2: Casein α S2
CSN2: Casein ß
CSN3: Casein κ
*E. coli*: *Escherichia coli*
FPKM: Fragments per kilobase per million fragments mapped
IGV: Integrative Genomics Viewer
IL6: Interleukin 6
IPA: Ingenuity pathway analysis
LALBA: α lactalbumin
PAEP: Progestogen-associated endometrial protein
PPP1CC: Protein phosphatase 1 catalytic subunit γ
RNA-Seq: RNA sequencing
RPS15A: Ribosomal protein S15a
rRNA: ribosomal RNA
RT-qPCR: Reverse transcriptase-quantitative polymerase chain reaction
TREM1: Triggering receptor expressed on myeloid cells 1
