# Supplementary figures and images for: Development and evaluation of a milk protein transcript depletion method for differential transcriptome analysis in mammary gland tissue
Source: BMC Genomics. 2019 May 22;20:400. doi: 10.1186/s12864-019-5781-3 (PMC6530097; doi:10.1186/s12864-019-5781-3)

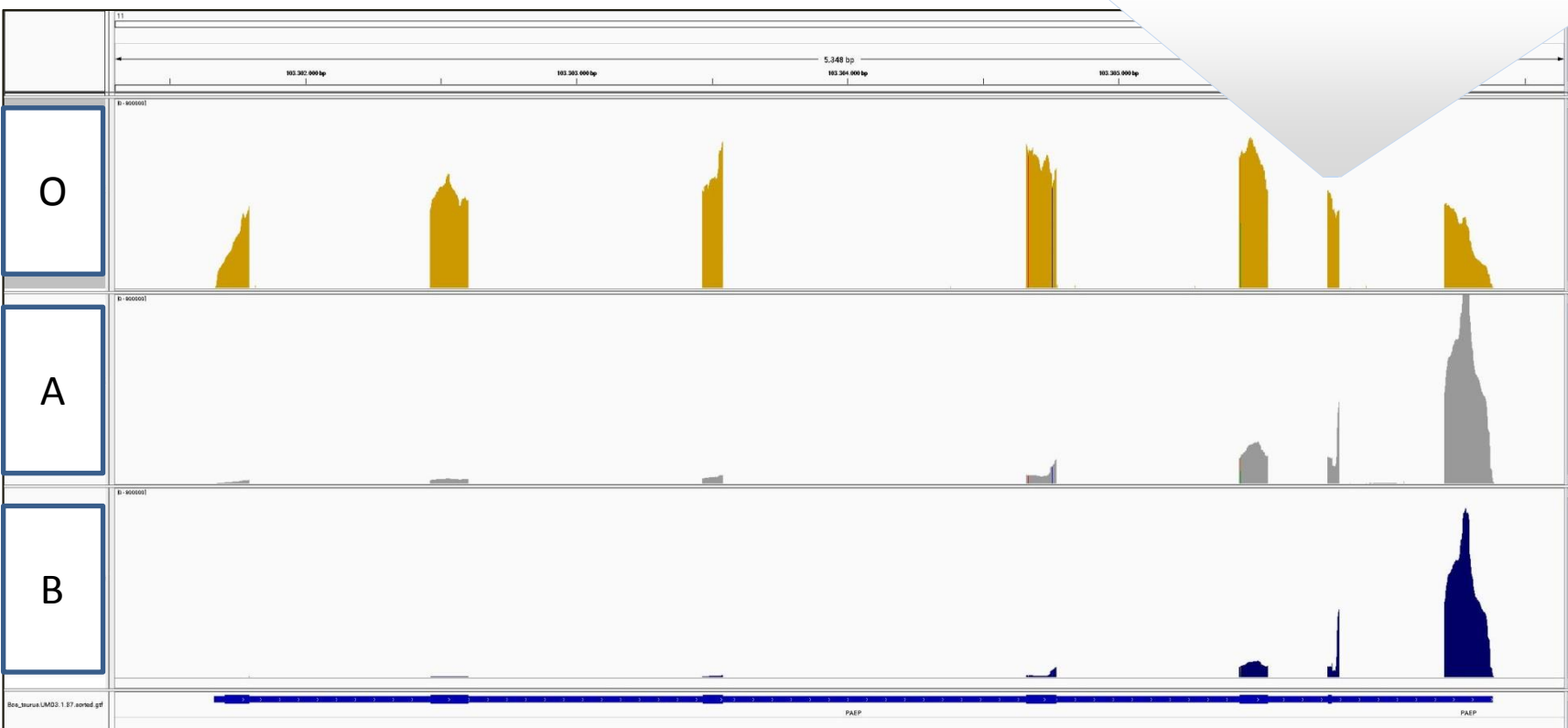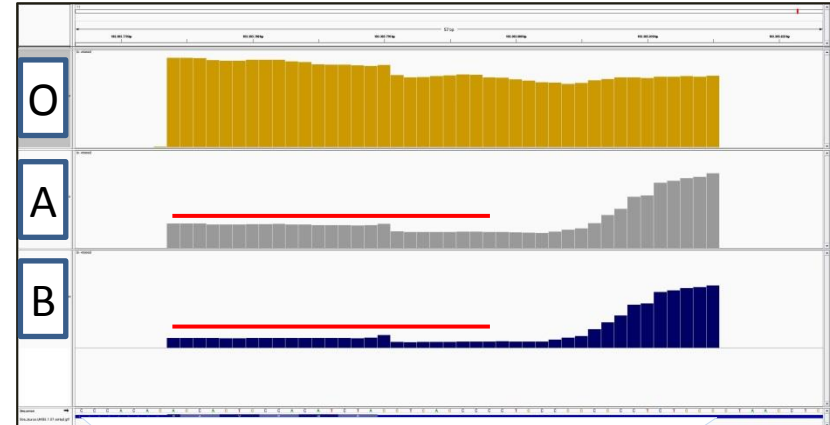

Supplement: Supplementary file 1 — IGV screen shot of the read distribution across the PAEP gene for the E. coli challenged udder sample from cow 2. O, A and B represent the non-depleted or the depleted RNA samples (protocol variant A and B), respectively. Exon 6 is zoomed in for better demonstration of the position of the antisense capture oligonucleotide (see red line). (PDF 235 kb) [file 12864_2019_5781_MOESM1_ESM.pdf]

## cow 1

challenged

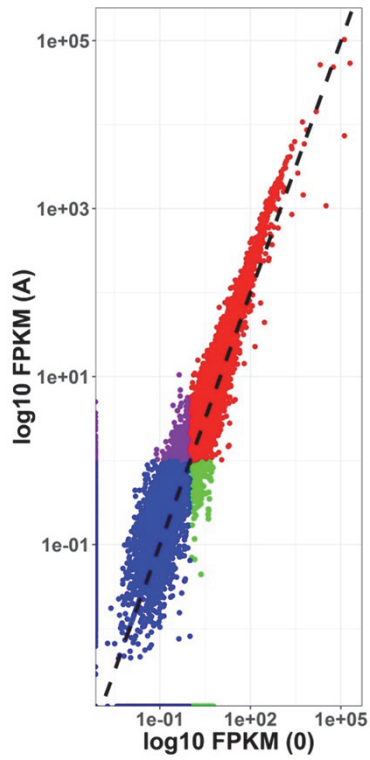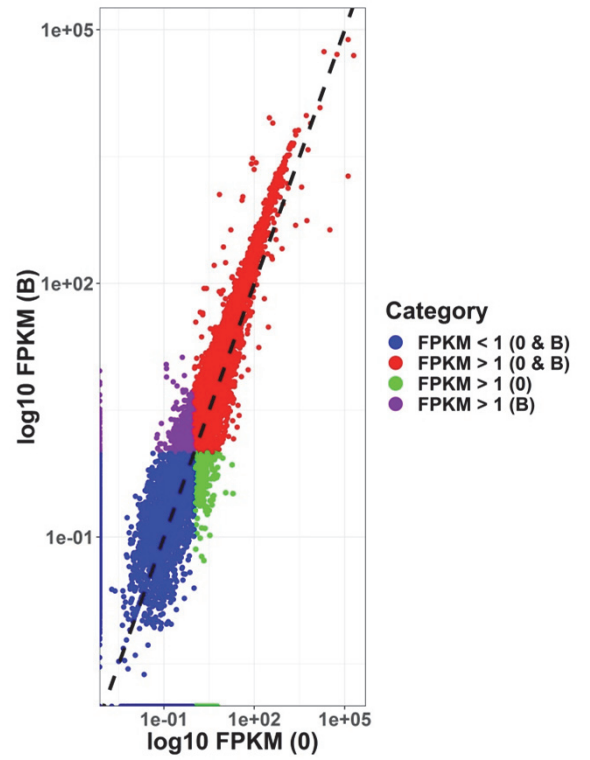

non-challenged

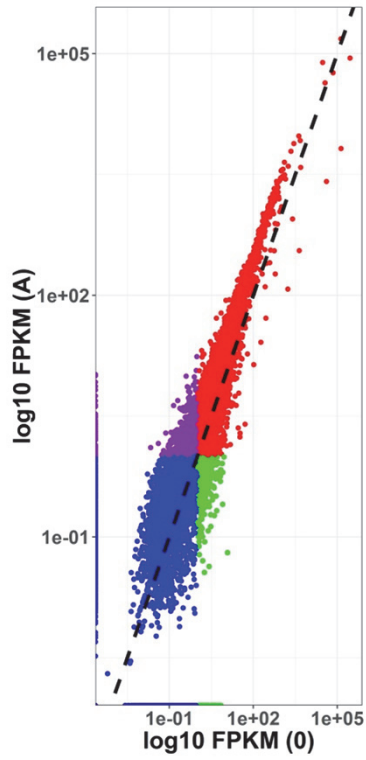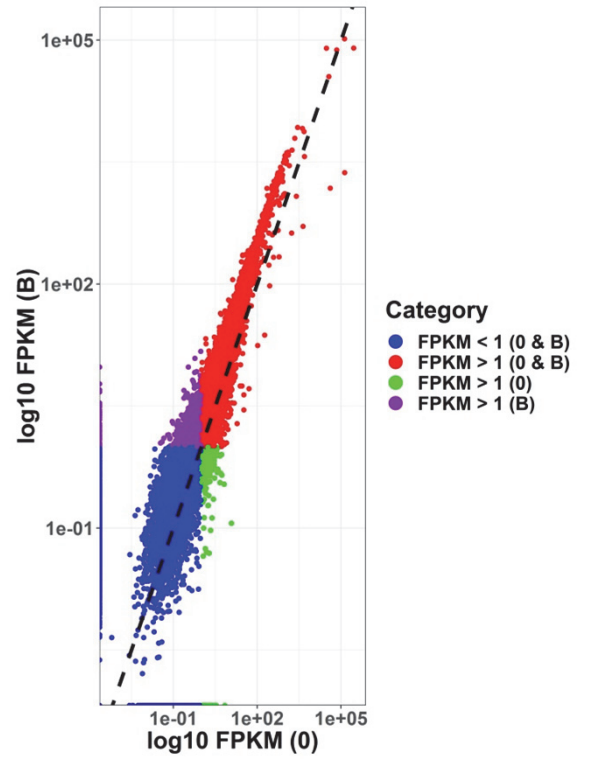

**cow 2**  
challenged

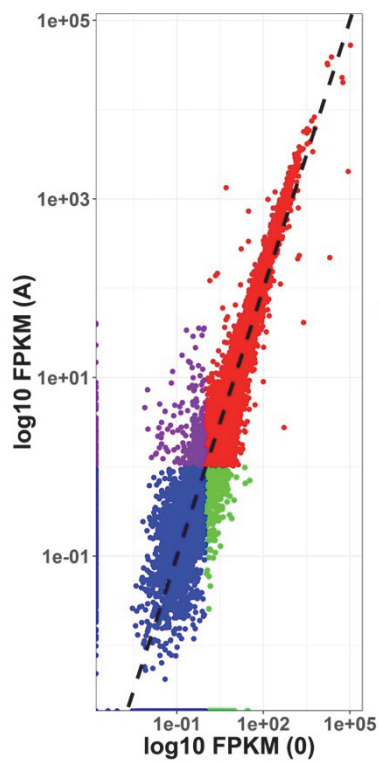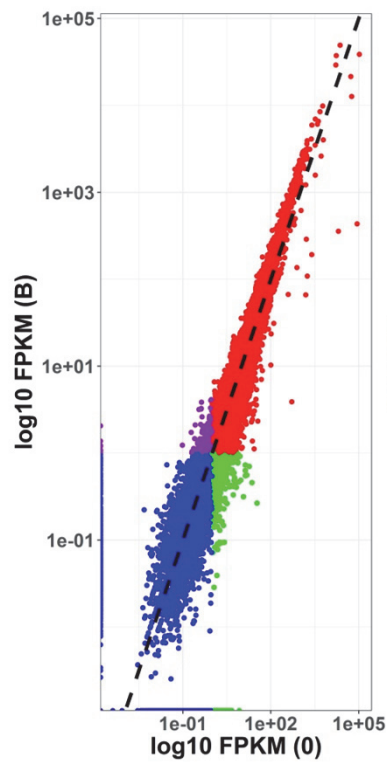

non-challenged

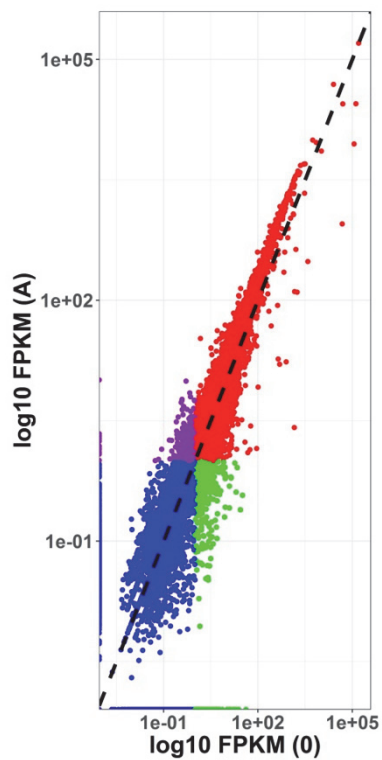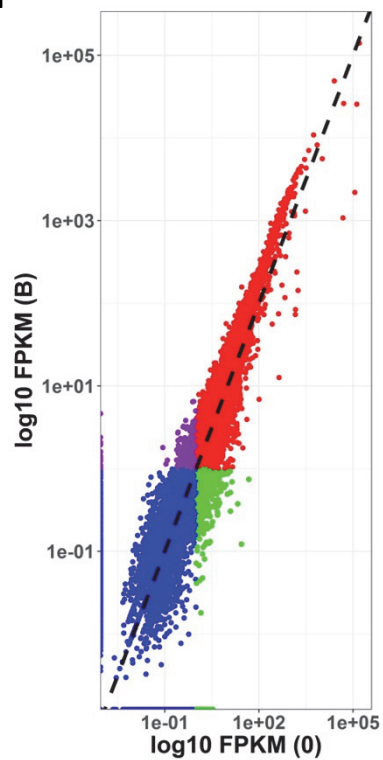

### cow 3

challenged

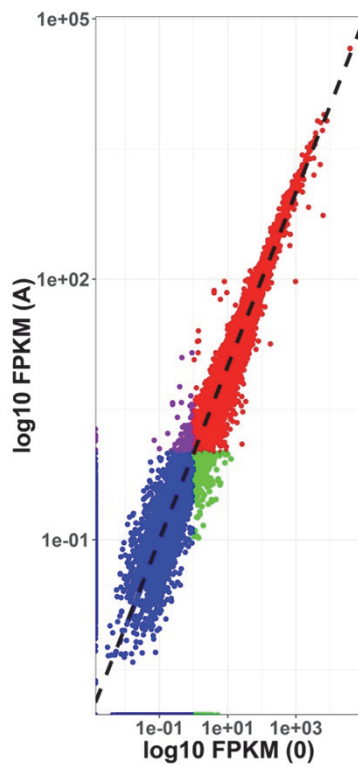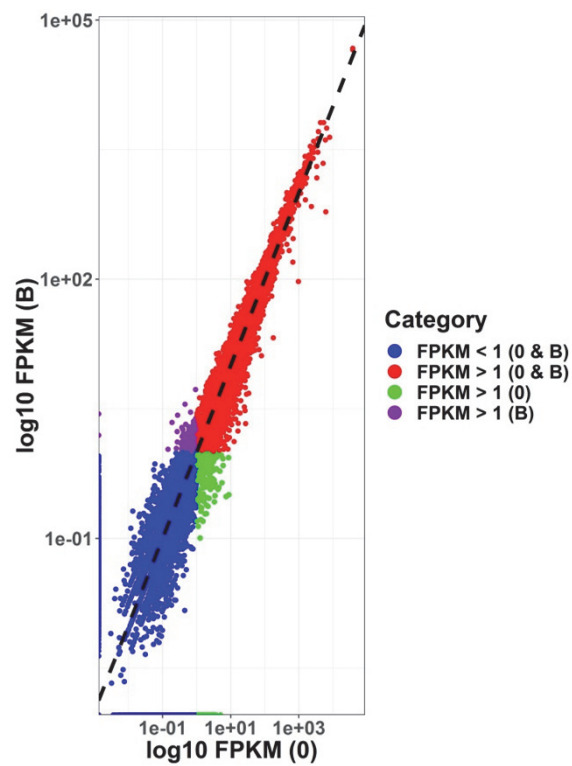

non-challenged

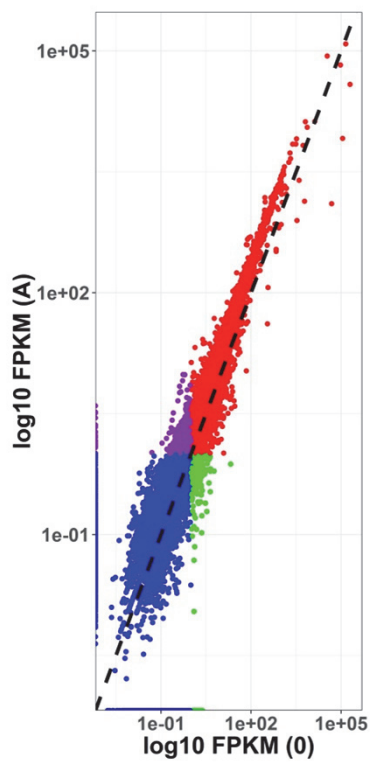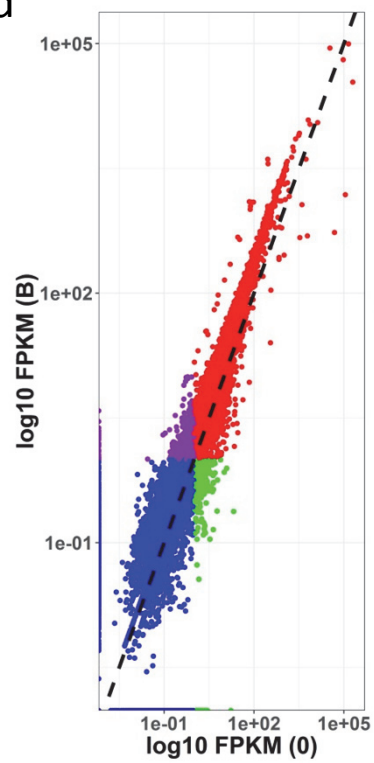

Supplement: Supplementary file 2 — Correlation plot of gene expression level (in log10 FPKM) between depleted and non-depleted RNA samples within E. coli challenged and non-challenged udder samples of each cow investigated. 0, A, and B represent the non-depleted or the depleted RNA samples (protocol variant A and B), respectively. (PDF 1551 kb) [file 12864_2019_5781_MOESM2_ESM.pdf]

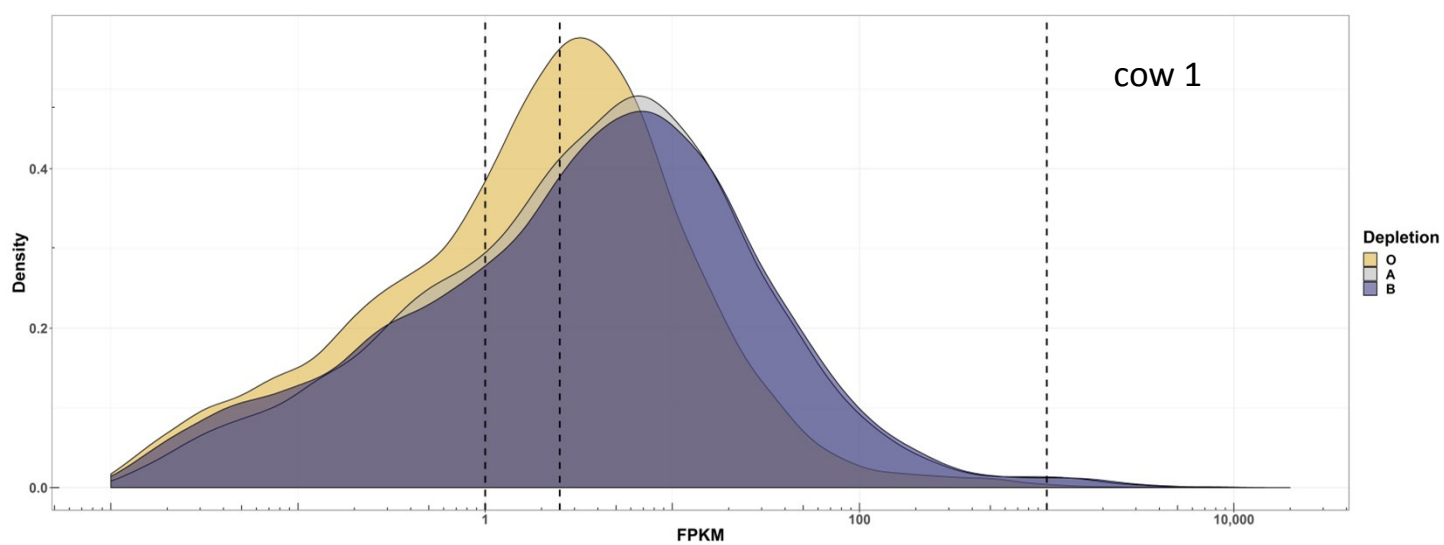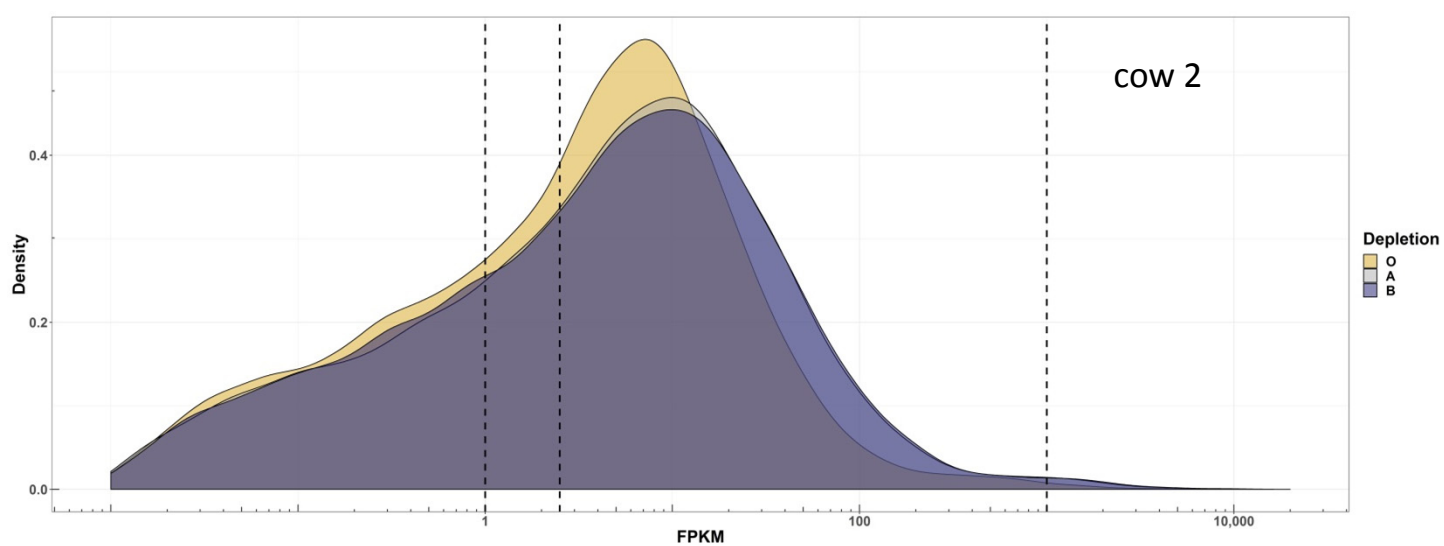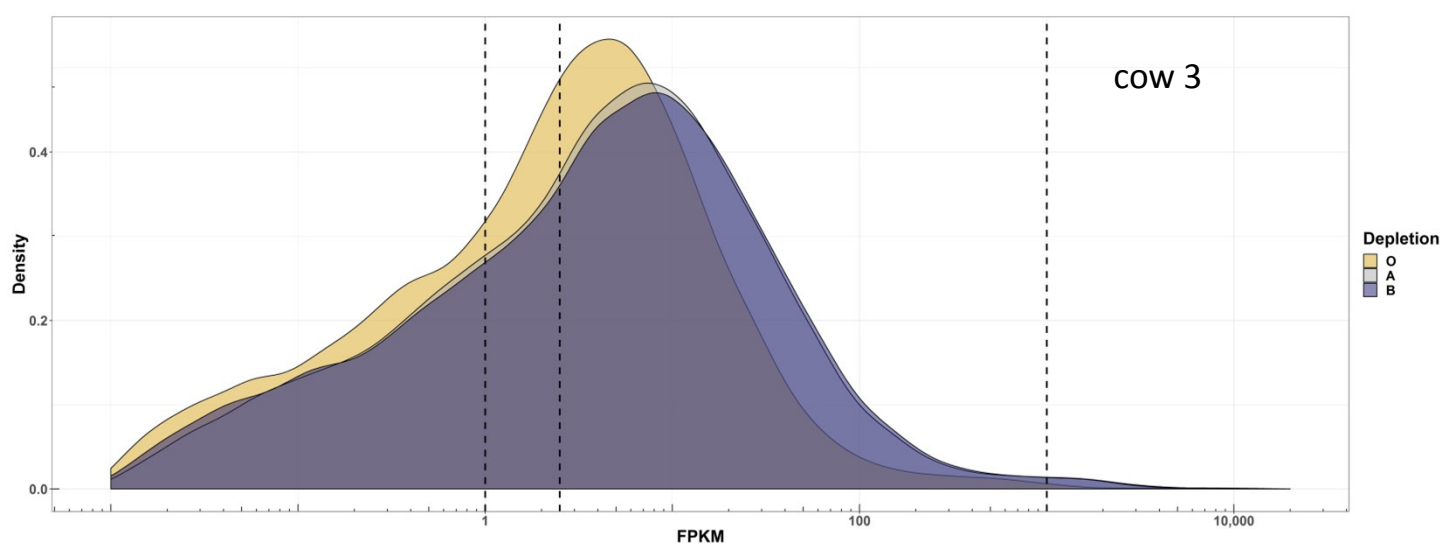

Supplement: Supplementary file 3 — Distribution of gene expression level (FPKM) for the depleted and non-depleted RNA samples from non-challenged udder samples. 0, A, and B represent the non-depleted or the depleted RNA samples (protocol variant A and B), respectively. (PDF 283 kb) [file 12864_2019_5781_MOESM3_ESM.pdf]

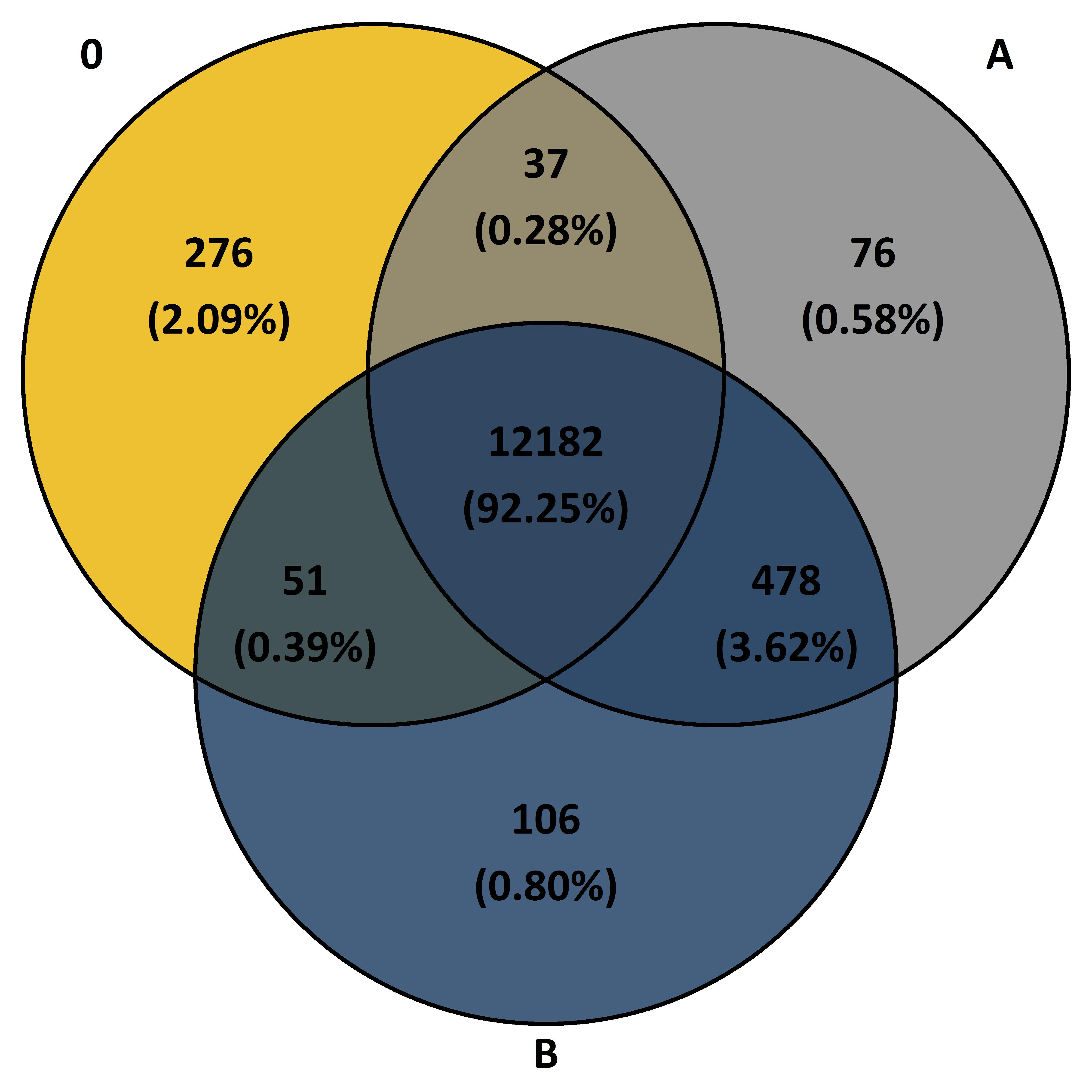

Supplement: Supplementary file 4 — Number and overlap of expressed genes with FPKM > 1 in depleted (protocol variants A and B) and non-depleted (0) RNA samples. (JPG 645 kb) [file 12864_2019_5781_MOESM4_ESM.jpg]
