# Supplementary material for: Development and evaluation of a milk protein transcript depletion method for differential transcriptome analysis in mammary gland tissue
Source: BMC Genomics. 2019 May 22;20:400. doi: 10.1186/s12864-019-5781-3 (PMC6530097; doi:10.1186/s12864-019-5781-3)
Supplement: Supplementary file 6 — Lists of IPA canonical pathways significantly enriched for differentially expressed genes in non-depleted (0) and depleted RNA samples (protocol variants A and B) from pathogen-challenged and non-challenged udder quarters. Pathways are classified for being observed across all protocols (0, A or B) or shared between two or all three protocols. (PDF 214 kb) [file 12864_2019_5781_MOESM6_ESM.pdf]

pathways identified with IPA

146 common elements in samples without depletion (0) and depletion (A and B):

4-1BB Signaling in T Lymphocytes
Actin Nucleation by ARP-WASP Complex
Activation of IRF by Cytosolic Pattern Recognition Receptors
Acute Phase Response Signaling
Agranulocyte Adhesion and Diapedesis
Agrin Interactions at Neuromuscular Junction
Altered T Cell and B Cell Signaling in Rheumatoid Arthritis
Antioxidant Action of Vitamin C
Apoptosis Signaling
April Mediated Signaling
Aryl Hydrocarbon Receptor Signaling
Atherosclerosis Signaling
Axonal Guidance Signaling
B Cell Activating Factor Signaling
B Cell Receptor Signaling
Caveolar-mediated Endocytosis Signaling
CD27 Signaling in Lymphocytes
CD28 Signaling in T Helper Cells
CD40 Signaling
CDP-diacylglycerol Biosynthesis I
Cell Cycle: G1/S Checkpoint Regulation
Cholecystokinin/Gastrin-mediated Signaling
Coagulation System
Colorectal Cancer Metastasis Signaling
Communication between Innate and Adaptive Immune Cells
Complement System
Crosstalk between Dendritic Cells and Natural Killer Cells
Death Receptor Signaling
Dendritic Cell Maturation
Differential Regulation of Cytokine Production in Intestinal Epithelial Cells by IL-17A and IL-17F
Differential Regulation of Cytokine Production in Macrophages and T Helper Cells by IL-17A and IL-17F
Eicosanoid Signaling
Endothelin-1 Signaling
ERK/MAPK Signaling
ERK5 Signaling
Erythropoietin Signaling
Estrogen-mediated S-phase Entry
Extrinsic Prothrombin Activation Pathway
Fatty Acid Activation
Fatty Acid β-oxidation I
Fcγ Receptor-mediated Phagocytosis in Macrophages and Monocytes
GADD45 Signaling
Germ Cell-Sertoli Cell Junction Signaling
G 12/13 Signaling
Gαq Signaling

Glioma Invasiveness Signaling
Glucocorticoid Receptor Signaling
G-Protein Coupled Receptor Signaling
Granulocyte Adhesion and Diapedesis
Hepatic Cholestasis
Hepatic Fibrosis / Hepatic Stellate Cell Activation
HGF Signaling
HMGB1 Signaling
gamma-linolenate Biosynthesis II (Animals)
iCOS-iCOSL Signaling in T Helper Cells
IL-1 Signaling
IL-10 Signaling
IL-12 Signaling and Production in Macrophages
IL-15 Production
IL-15 Signaling
IL-17A Signaling in Airway Cells
IL-17A Signaling in Fibroblasts
IL-17A Signaling in Gastric Cells
IL-6 Signaling
IL-8 Signaling
IL-9 Signaling
ILK Signaling
Induction of Apoptosis by HIV1
Inflammasome pathway
Inhibition of Matrix Metalloproteases
iNOS Signaling
Integrin Signaling
Interferon Signaling
Iron homeostasis signaling pathway
JAK/Stat Signaling
Leukocyte Extravasation Signaling
LPS/IL-1 Mediated Inhibition of RXR Function
LPS-stimulated MAPK Signaling
LXR/RXR Activation
Lymphotoxin beta Receptor Signaling
MIF Regulation of Innate Immunity
MIF-mediated Glucocorticoid Regulation
Mitochondrial L-carnitine Shuttle Pathway
Molecular Mechanisms of Cancer
MSP-RON Signaling Pathway
Neuroinflammation Signaling Pathway
NF-κB Activation by Viruses
NF-κB Signaling
Oncostatin M Signaling
Opioid Signaling Pathway
Osteoarthritis Pathway
p38 MAPK Signaling
Pancreatic Adenocarcinoma Signaling
Paxillin Signaling
Phagosome Formation

Phosphatidylglycerol Biosynthesis II (Non-plastidic)
Phospholipase C Signaling
PI3K Signaling in B Lymphocytes
PI3K/AKT Signaling
PKC theta¸ Signaling in T Lymphocytes
PPAR Signaling
PPARα/RXRα Activation
Primary Immunodeficiency Signaling
Production of Nitric Oxide and Reactive Oxygen Species in Macrophages
PTEN Signaling
RANK Signaling in Osteoclasts
Regulation of Actin-based Motility by Rho
Regulation of IL-2 Expression in Activated and Anergic T Lymphocytes
Relaxin Signaling
Role of Hypercytokinemia/hyperchemokinemia in the Pathogenesis of Influenza
Role of IL-17A in Arthritis
Role of JAK family kinases in IL-6-type Cytokine Signaling
Role of JAK1, JAK2 and TYK2 in Interferon Signaling
Role of Macrophages, Fibroblasts and Endothelial Cells in Rheumatoid Arthritis
Role of MAPK Signaling in the Pathogenesis of Influenza
Role of NFAT in Regulation of the Immune Response
Role of Osteoblasts, Osteoclasts and Chondrocytes in Rheumatoid Arthritis
Role of Pattern Recognition Receptors in Recognition of Bacteria and Viruses
Role of PI3K/AKT Signaling in the Pathogenesis of Influenza
Role of PKR in Interferon Induction and Antiviral Response
Role of RIG1-like Receptors in Antiviral Innate Immunity
Role of Tissue Factor in Cancer
Semaphorin Signaling in Neurons
Sertoli Cell-Sertoli Cell Junction Signaling
Signaling by Rho Family GTPases
Sirtuin Signaling Pathway
Small Cell Lung Cancer Signaling
STAT3 Pathway
T Cell Receptor Signaling
T Helper Cell Differentiation
Tec Kinase Signaling
Th1 and Th2 Activation Pathway
Th1 Pathway
Th2 Pathway
TNFR1 Signaling
TNFR2 Signaling
Toll-like Receptor Signaling
TREM1 Signaling
Triacylglycerol Biosynthesis
Tumoricidal Function of Hepatic Natural Killer Cells
TWEAK Signaling
Type I Diabetes Mellitus Signaling
Type II Diabetes Mellitus Signaling
VDR/RXR Activation
Virus Entry via Endocytic Pathways

Wnt/β-catenin Signaling

12 common elements in samples without depletion (0) and samples depleted with variant A:
Acetate Conversion to Acetyl-CoA
Adipogenesis pathway
Ceramide Signaling
Ephrin Receptor Signaling
GNRH Signaling
Intrinsic Prothrombin Activation Pathway
Rac Signaling
RAR Activation
Regulation of Cellular Mechanics by Calpain Protease
Renin-Angiotensin Signaling
RhoGDI Signaling
Sphingosine-1-phosphate Signaling

8 common elements in samples without depletion (0) and samples depleted with variant B:
Circadian Rhythm Signaling
Histidine Degradation VI
IL-17 Signaling
Pregnenolone Biosynthesis
Role of JAK1 and JAK3 in γc Cytokine Signaling
Stearate Biosynthesis I (Animals)
Sumoylation Pathway
Xenobiotic Metabolism Signaling

6 common elements in samples depleted with variant A and B:
Clathrin-mediated Endocytosis Signaling
Gαs Signaling
Graft-versus-Host Disease Signaling
Reelin Signaling in Neurons
Regulation of the Epithelial-Mesenchymal Transition Pathway
Role of IL-17F in Allergic Inflammatory Airway Diseases

8 elements included exclusively in samples depleted with variant B:
Airway Pathology in Chronic Obstructive Pulmonary Disease
Chondroitin Sulfate Degradation (Metazoa)
Fc Epsilon RI Signaling
GP6 Signaling Pathway
Granzyme A Signaling
Role of IL-17A in Psoriasis
Role of JAK2 in Hormone-like Cytokine Signaling
Ubiquinol-10 Biosynthesis (Eukaryotic)

9 elements included exclusively in samples depleted with variant A:
autophagy
B Cell Development
Ethanol Degradation II
Ethanol Degradation IV
fMLP Signaling in Neutrophils

Gluconeogenesis I
nNOS Signaling in Skeletal Muscle Cells
Oxidative Ethanol Degradation III
Systemic Lupus Erythematosus Signaling

7 elements included exclusively in samples without depletion (0):

Angiopoietin Signaling
FXR/RXR Activation
Glioblastoma Multiforme Signaling
Glycolysis I
OX40 Signaling Pathway
Protein Kinase A Signaling
Thrombin Signaling
